# Image feature embedding with a deep learning framework improves genome-wide association studies on dog endophenotypes

**DOI:** 10.1126/sciadv.aee1088

**Published:** 2026-06-24

**Authors:** Guang-Xiao E, Guo-Dong Wang

**Affiliations:** ^1^ School of Life Sciences, Yunnan University, Kunming 650500, China.; ^2^ State Key Laboratory of Genetic Evolution & Animal Models, Genome Center of Biodiversity, Kunming Institute of Zoology, Chinese Academy of Sciences, Kunming 650201, China.

## Abstract

Domestic dogs exhibit substantial morphological diversity, making quantitative characterization of their phenotypes challenging. Traditional phenotyping methods often rely on manual measurements, which are limited in their ability to capture complex visual traits. Deep learning provides an opportunity to automatically extract informative and biologically meaningful features from images. In this study, we constructed a dataset of 13,254 dog images across multiple breeds and used ResNet and ViT models to automatically extract 256-dimensional image embeddings. After dimensionality reduction using UMAP (uniform manifold approximation and projection), we performed a GWAS (genome-wide association study) on the extracted features and breed-level genotype data. We identified 15 genes previously reported to be associated with dog traits such as hair length and body size, as well as previously unknown candidate genes related to body development and hair growth, including *EIF2S2*, *TRHR*, and *TCF25*, which harbor variants with potential functional relevance. This approach is validated by known genetic associations and can reveal previously unidentified genotype-phenotype links. Building on these capabilities, this approach provides a scalable framework for phenotype extraction that enables population genetic studies in domestic dogs and can facilitate breeding in other economically important species.

## Teaser

Deep learning image embeddings enable scalable GWAS of complex dog phenotypes.

## INTRODUCTION

Domestic dogs (*Canis lupus familiaris*), as the earliest domesticated animals by humans, have undergone intense artificial selection as well as natural selection over tens of thousands of years (*1*, *2*). Genome-wide association studies (GWASs) and whole-genome sequencing–based selective sweep analyses have proven to be powerful complementary tools for identifying the genetic basis of these traits. By combining whole-genome sequencing and standard breed information for each breed from the American Kennel Club (AKC; akc.org/dog-breeds/) (*3*), genes that control variation traits between breeds were identified, among which only 14 genes can explain 95% of the breed-average weight variation in purebred dogs (*4*). Previous studies have provided detailed reports on the genes of dogs on the basis of abundant manual annotations of phenotypes and have successfully identified a large number of genes underlying key traits, such as coat color, coat length, and body size (*4*–*7*). This rich understanding of canine traits, together with their shared phenotypes with humans and high disease-sharing rate, has positioned dogs as an ideal model for studying human diseases (*8*–*11*).

Domestic dogs exhibit a genetic structure at multiple levels. During the earliest stages of domestication, selection primarily targeted adaptive traits such as dietary preferences (*12*, *13*) and aggression levels (*14*). In later stages of development, artificial selection became more focused on specific behavioral and morphological traits (*15*, *16*). Breeders have historically crossed dogs within specific groups to enhance their behavioral traits (e.g., defense and herding) and morphology (e.g., coat color and coat length) (*17*–*20*). The domestication history has reduced the phenotypic differences between breeds and linkage disequilibrium (LD) within breeds (*21*).

Expert scoring and measurement of breed traits are often the first choice for phenotypic data acquisition. However, these traditional approaches rely heavily on predefined and manually measured traits, which may oversimplify the complex and multidimensional variation among dog breeds. Subtle morphological differences, behavioral characteristics, and other less visually obvious traits are often overlooked, and the inherent coding schemes used for genetic mapping cannot fully capture the richness and continuity of these phenotypes. In contrast, descriptive features from deep learning are often derived through the integration of information directly from pixel-level data (*22*). In medical image tasks, deep learning models can be used to extract organ-level image features as quantitative representations of patients’ endophenotypes for GWAS, which allows these data-driven imaging biomarkers to be associated with genetic variants across the genome and has led to the successful identification of genetic loci underlying the morphology and pathology of ocular structures, brain anatomy, and vascular tissue (*23*–*25*). Building on these advances, we believe that deep learning–based image recognition applied to domestic dog images can similarly capture meaningful phenotypic representations, providing effective quantitative variables for GWAS to elucidate the genetic architecture of canine morphological diversity.

In the present study, we aimed to integrate image-based phenotypic feature embedding with a GWAS to explore the genetic basis of morphological variation in dogs. We first collected 13,254 images of dogs from 181 breeds by querying commercial search engines to train residual network (ResNet) (*26*) and vision transformer (ViT) (*27*) image classification models. We then extracted high-dimensional features from these trained models and used uniform manifold approximation and projection (UMAP) to project these embeddings into a lower-dimensional space as phenotypic data for the GWAS. These features serve as continuous and quantitative endophenotypes, providing image-derived phenotypic representations for the Dog10K genotypic dataset, effectively capturing the overall visual morphological differences between breeds to identify genetic loci governing these appearance traits, especially the hair between dog breeds.

## RESULTS

### Image dataset preparation and feature embedding

**Fig. 1.**
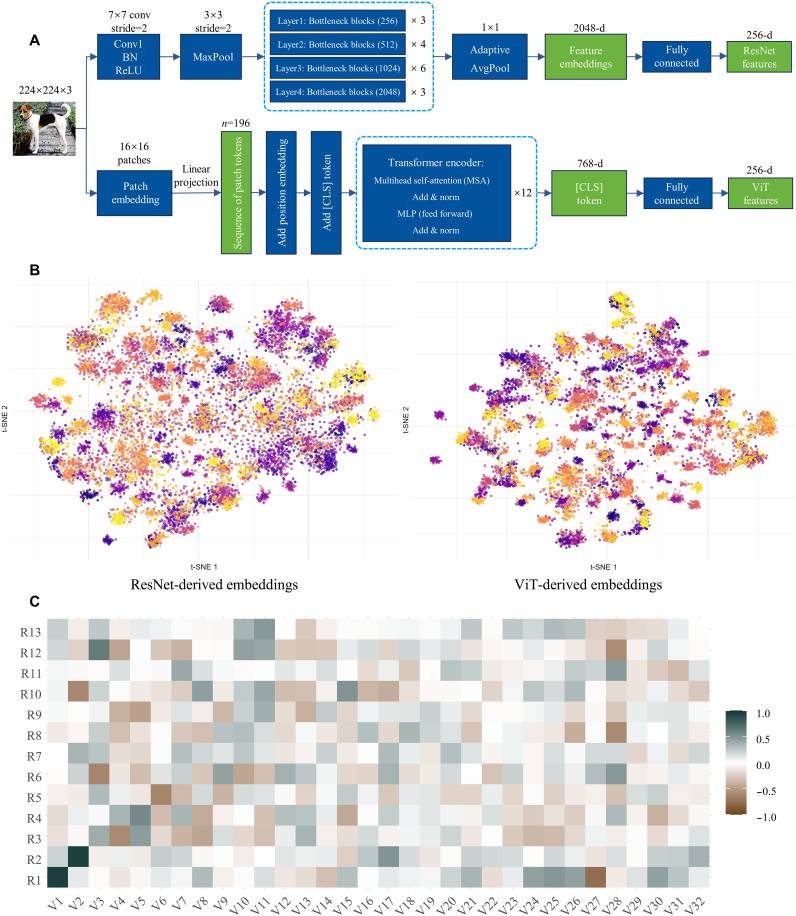
Image feature embedding via fine-tuned, trained models and dimensionality reduction. (**A**) Workflow of image feature embedding. ResNet and ViT were used as backbone networks for feature extraction. The fully connected and softmax layers of ResNet, as well as the classification head of ViT, were removed. A fully connected layer with an output dimension of 256 was added at the end of each network. The extracted embeddings were saved as a 256-dimensional feature. (**B**) t-SNE dimensionality reduction visualization. The different colors indicate the groups of the data points. (**C**) Heatmap of the correlations between ResNet embedding features (R1 to R13) and ViT embedding features (V1 to V32). Image credit: Wikimedia Commons (see table S1 for details).

We first constructed an image dataset. Specifically, we collected 13,254 images of 181 breeds through the Bing search engine. After filtering, we obtained a high-quality image dataset suitable for analysis. For feature extraction, we used two widely used deep learning architectures, ResNet and ViT, representing the classical approaches in convolutional and transformer-based visual representation learning, respectively. We modified the classification layer in both models to output a 256-dimensional embedding feature for each image. This approach facilitates a direct comparison between the models and enables downstream tasks (Fig. 1A). We further validated model attention using Grad-CAM by comparing activations in dog and background regions to improve the spatial interpretability of layer-wise representations. The results revealed that the activation intensities were substantially greater in the dog regions for 99.08% of the ResNet images and 93.98% of the ViT images. These findings indicated that the learned embeddings predominantly capture biologically relevant features rather than background cues.

To qualitatively assess the effectiveness of the extracted features, we first visualized the 256-dimensional embeddings using t-distributed stochastic neighbor embedding (t-SNE). Visualization reveals clear clustering patterns, with images of the same dog breed forming compact and well-separated groups (intra/interclass distance ratio: ResNet = 0.266, ViT = 0.215), suggesting that both ResNet and ViT encode relevant semantic information in their latent representations. To further explore these high-dimensional embeddings, we used UMAP, a nonlinear dimensionality reduction framework that not only enables direct visualization of raw image data (*28*) but also preserves the intrinsic clustering structure of features extracted from neural networks and reveals the intrinsic relational patterns among them (*29*). By comparing the embedding spaces of ResNet and ViT, we can analyze how each model structures semantic information and whether one provides a better clustering effect than the other does. ViT, which retains more spatial location information in its higher layers (*30*), appears to extract richer features, resulting in t-SNE visualizations with clearer interbreed clustering patterns (Fig. 1B). This richness is further supported by UMAP analysis, which revealed that the ViT embeddings require more dimensions (*n* = 32) to fully capture structural information, whereas ResNet embeddings need fewer dimensions (*n* = 13), indicating that ViT encodes more complex and informative representations (figs. S1 and S2).

Although UMAP is a nonlinear dimensionality reduction technique and does not produce explicit variance ratios like principal components analysis (PCA), the variance between breeds in the UMAP-reduced dimensions still reflects the variance retained from the original data. When UMAP is applied with Laplacian eigenmap (LE) initialization, the variance becomes significantly larger in the first few dimensions, whereas using LE alone results in a relatively uniform variance distribution across dimensions (fig. S3). UMAP with LE initialization preserves the global, low-frequency structure captured by LE, as illustrated by the breed-averaged correlation heatmaps presented, and effectively clusters images across breeds (figs. S4 and S5).

By first calculating the mean feature values within each breed and then performing correlation analysis between the features of the two models, we can quantify their similarity and evaluate whether both models capture similar semantic information. The highest Pearson correlation is exhibited in R1-V1 (*r* = 0.95, *P* < 0.001) and R2-V2 (*r* = 0.94, *P* < 0.001), indicating that they identified shared traits in the first two features. Because the low-frequency structure preserved by the LE initialization of UMAP reflects smooth, global variation across samples, these features are likely to represent the main distinguishing information and capture major crossbreed visual phenotypic differences. The follow-up highest correlation features indicate that the two models diverge in how they rank the importance of the remaining features, suggesting that although both models consistently capture the dominant low-frequency structure of the data, they differ in their representation of higher-frequency variations (Fig. 1C).

### Significant correlation between embedding features and phenotype

**Fig. 2.**
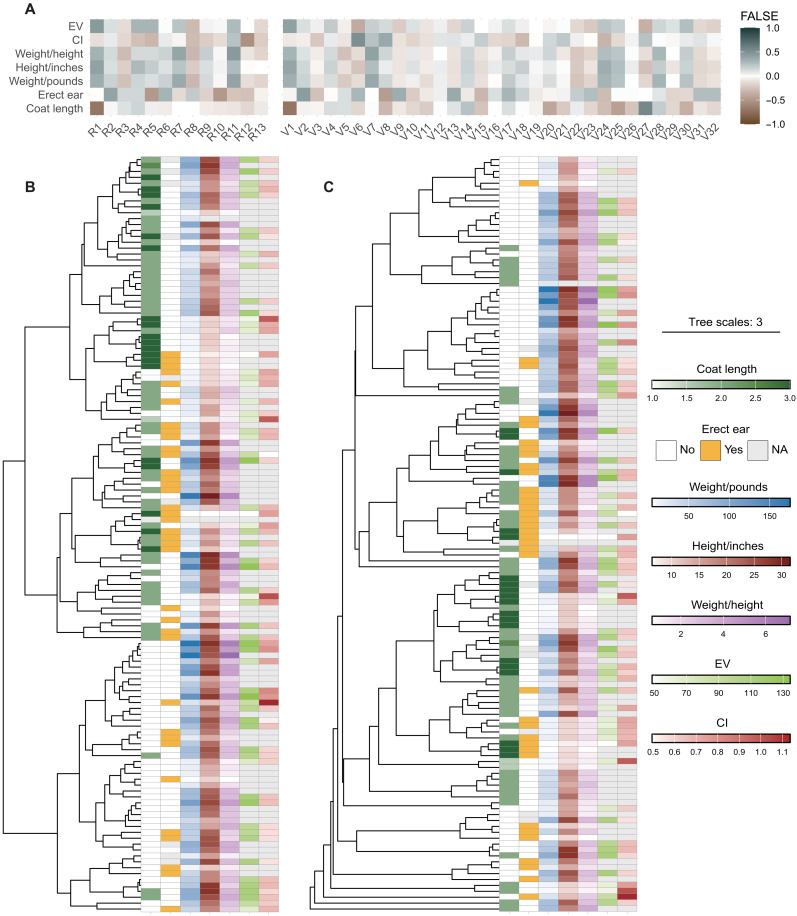
Correlation between image-derived features and breed phenotypes and hierarchical clustering based on image embeddings. (**A**) Correlation heatmap between embedding features and phenotypes of dog breeds: coat length, erect ears, weight, height, weight/height, CI, and EV. (**B**) Hierarchical clustering tree constructed from ResNet. (**C**) Hierarchical clustering tree constructed from ViT.

To further explore the biological significance of the embedded features, we quantified the correspondence between the breed-averaged embedding features and phenotypic traits via Pearson correlation. We used phenotypic data from the AKC and peer-reviewed sources, as compiled in a previous study, encompassing 20 phenotypic traits across 149 dog breeds (e.g., body size, lifespan, snout ratio, and shedding) (*31*). In addition, we incorporated cranial morphometric data from 1682 individual dogs representing 172 breeds (*32*). We computed the average embedding representation for each breed and kept the intersection of the phenotype files of breeds and 181 breeds. From the above explanation, feature 1 is the most important feature across both models and is highly correlated (R1: *r* = 0.95, *P* < 0.001), and the models share similarity in real phenotype correlation, which is highly correlated with coat length in dog images (R1: *r* = −0.7, *P* < 0.001; V1: *r* = −0.7, *P* < 0.001). When the fine-grained fur texture was progressively suppressed through region-level superpixel masking, the correlation between the embedding features and coat length decreased with increasing superpixel compactness in both models (features most strongly associated with coat length at compactness levels 40, 60, and 80: ResNet: R3 = −0.56, *P* < 0.001; R3 = 0.40, *P* < 0.001; R2 = 0.37, *P* < 0.001; ViT: V1 = 0.52, *P* < 0.001; V20 = −0.47, *P* < 0.001; V32 = 0.35, *P* < 0.001). These results provide additional evidence that the learned representations capture biologically meaningful visual cues rather than spurious background information. Erect ears are associated with multiple features (R2: *r* = 0.39, *P* < 0.001; R5: *r* = −0.48, *P* < 0.001; R10: *r* = −0.48, *P* < 0.001; V2: *r* = 0.43, *P* < 0.001; V8: *r* = −0.46, *P* < 0.001) (Fig. 2A), but owing to their relatively low correlations, these features may also capture other undefined phenotypic variations. The phenotypic tree separated the breeds into two distinct clusters primarily driven by coat length, while weaker clustering patterns were observed for erect ears, body size–related traits, cranial index (CI), and endocranial volume (EV) (Fig. 2, B and C, and table S2).

Significant correlations were observed between model-derived embedding dimensions and phenotypic traits, with the first UMAP feature in particular aligning with major morphological variations such as body size and coat length. Moreover, the phenotypic tree constructed from the embedding features revealed meaningful clustering patterns, including the separation of short-haired or hairless breeds and the grouping of erect ear breeds, demonstrating that the embedding features are biologically meaningful and discriminative. The associations and clustering patterns provided strong motivation to conduct a GWAS, aiming to identify genetic variants underlying the phenotypic differences reflected in the embedding features.

### ResNet embedding analyses

We performed a GWAS using 13 embedding features as phenotypes. On the basis of the genomic inflation factor and the histogram of single-nucleotide polymorphism (SNP) *P* value distributions (fig. S6), we observed that as the embedding dimension increases, the proportion of variance tends to decrease, with the valley variance values often corresponding to greater deviations of λ_GC_ from 1.

As expected, feature 1, which is associated with coat length, resulted in the identification of several significant genes, including polycystic kidney and hepatic disease 1 (*PKHD1L1*), oxidation resistance 1 (*OXR1*), R-spondin 2 (*RSPO2*), eukaryotic translation initiation factor 3 subunit E (*EIF3E*), cilia- and flagella-associated protein 299 (*CFAP299*), fibroblast growth factor 5 (*FGF5*), and angiopoietin 1 (*ANGPT1*). We also identified genes that have been reported in other mammals. For example, *CFAP299* and *FGF5* were previously reported to be located in a shared strong selection region in Tianzhu White Yak. Notably, *FGF5* encodes a fibroblast growth factor that regulates hair length and type across multiple species, including dogs, long-haired mice, cats, donkeys, and goats (*33*–*36*). In this study, its strongest association signal was identified at CFA32: 35494497 (*P* < 1 × 10^−31^), corresponding to a Cys^95^→Phe (C95F) substitution, which has been previously validated for its functional effect (*5*). In addition, other genes, such as *PKHD1L1*, *OXR1*, *EIF3E*, and *RSPO2*, have been implicated in the regulation of long hair (*7*, *37*). The *ANGPT1* gene is important for controlling both angiogenesis and muscle development, and an increase in its copy number is significantly correlated with carcass weight and body weight in goats (*38*). In addition to the aforementioned genes, feature 2 is β-defensin 103 (*CBD103*), which affects hair color in various canids (*39*, *40*). It encodes a β-defensin that interacts with *MC1R* to regulate coat color in dogs. Its dominant black allele (*K^B^*) activates *MC1R* signaling, producing black pigment and overriding Agouti’s inhibition, which would otherwise lead to yellow coats (*41*). The appearance of this gene in the second dimension suggests that coat color is an important factor in distinguishing breeds.

In the subsequent GWAS results using the following features, various aspects of detailed traits were associated with multiple SNPs (Table 1). *MSRB3* and *HMGA2* control the ear type and body size (*42*–*45*) of multiple mammals (R4 and R7). The color pattern genes melanocyte-inducing transcription factor (*MITF*) (R4, R5, and R6) and heterogeneous nuclear ribonucleoprotein associated with lethal yellow (*RALY*) (R4) were strongly associated (*46*, *47*). The GWAS results for the first few features typically showed clear and prominent peaks, whereas those for the later features tended to display more scattered signals, with SNPs appearing less clustered.

**Table 1.** Genes identified by ResNet embedding.

Feature	Symbol	Position/region	Function	Association
1	*PKHD1L1-OXR1-RSPO2-EIF3E*, *ANGPT1*, *TRHR*	chr13: 7295774–10242164	Multiple functions	Hair length (*7*, *37*) and body development (*38*, *66*)
	*SEMA3C*	chr18: 20744421–20890125	Encodes secreted glycoprotein	Distraction index (*37*)
	*CFAP299-FGF5*	chr32: 34864306–35498419	Cilia- and flagella-associated protein and fibroblast growth factor (*33*)	Hair length (*33*)
2	*PKHD1L1-OXR1-RSPO2-EIF3E*, *ANGPT1*	chr13: 7295774–10176662	Multiple functions	Hair length and body development
	*CBD103*	chr16: 55464290–55473955	Antibiotic peptide (*40*)	Hair color (*39*, *40*)
	*HPCAL1*	chr17: 7330296–7331184	Calcium homeostasis (*99*)	Conformation traits (*100*)
	*HTT*	chr3: 61753985–61753985	Huntington disease protein (*101*)	Huntington’s disease (*101*)
4	*MSRB3-HMGA2*	chr10: 8332064–8797015	Multiple functions	Body size (*44*, *45*) and ear type (*42*, *43*)
	*CBD103*	chr16: 55463826–55473955	Antibiotic peptide	Hair color
	*SEMA3C*	chr18: 20744421–20890125	Encodes secreted glycoprotein	Distraction index
	*MITF*	chr20: 22053939–22064179	Regulates melanocyte development (*47*)	Spotting patterns in dogs (*47*)
	*RALY*-*ASIP*	chr24: 23706988–23906214	Multiple functions	Saddle tan, black-and-tan phenotypes (*46*) and black coat (*70*)
5	*MITF*	chr20: 21996459–22079634	Regulates melanocyte development	Spotting patterns in dogs
6	*CBD103*	chr16: 55473955–55473955	Antibiotic peptide	Hair color
	*MITF*	chr20: 21996459–22136145	Regulates melanocyte development	Spotting patterns in dogs
	*FGF5*	chr32: 35494497–35494497	Fibroblast growth factor	Hair length
7	*HMGA2*	chr10: 8746468–8768425	Transcriptional regulating factor (*102*)	Body type

### Feature alignment between ResNet and ViT

**Fig. 3.**
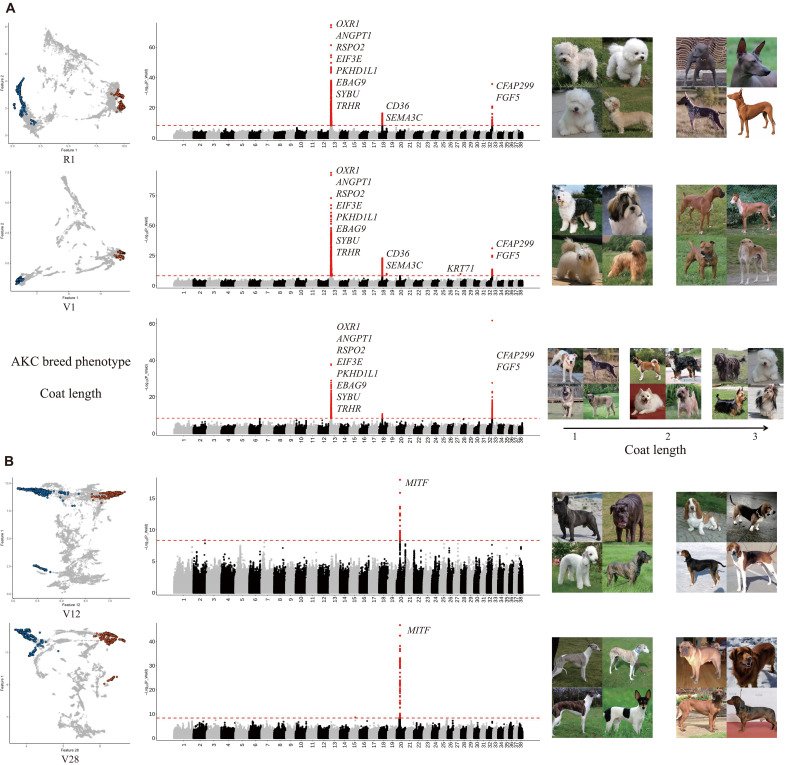
UMAP visualizations, Manhattan plots, and representative breed images for selected embedding features. Manhattan plots of the GWAS results, UMAP scatterplots highlighting the four breeds with the highest (orange) and lowest (blue) feature values, and representative breed images. To generate endophenotypes for GWAS, features were randomly assigned from the breed-level embedding feature set to individual genomic samples of the corresponding breed. (**A**) Results for embedding features R1 and V1, together with breed-level coat lengths from the AKC, assigned uniformly to all individuals within each breed. (**B**) Results for embedding features R12 and V28. Image credit: Wikimedia Commons (see table S1 for details).

The most prominent peak corresponding to feature 1 corresponds to coat-related genes, which is corroborated by the strong correlation between ViT feature 1 and ResNet feature 1 (*r* = 0.95, *P* < 0.001) and its high correlation with coat length (*r* = 0.7, *P* < 0.001). A GWAS performed using AKC-recorded breed coat length revealed highly overlapping association signals with those identified from feature 1, providing independent validation that this image-derived feature captures biologically meaningful variation in coat length (Fig. 3A and fig. S7). Both can separate breeds with different hair lengths; the breeds with the lowest feature 1 are long-haired dogs (Shih Tzu, Bolognese dog, and Bichon Frise), and the breeds with the greatest feature 1 are short-haired or hairless dogs (Xoloitzcuintli, Boxer, and Peruvian Hairless). The difference is that ViT located keratin 71 (*KRT71*) and fibroblast growth factor 4 (*FGF4*). *KRT71* variants cause a curly coat phenotype in rats, dogs, and cats (*5*, *48*, *49*). *FGF4* participates in the formation of hair ridges, and its retrogene causes chondrodystrophy in dogs (Fig. 3A) (*50*, *51*). Although there are low correlations with coat length for R2 (*r* = 0.02, *P* = 0.81) and V2 (*r* = −0.04, *P* = 0.70), a high correlation (*r* = 0.94, *P* < 0.001) occurs between them; a list of coat phenotype genes is also located in feature 2 of both models (*PKHD1L1*, *OXR1*, *RSPO2*, and *EIF3E*) (Table 2), and these genes have the best peak signal in the Manhattan plot. The second smoothness eigenvector in the LE may focus on other coat phenotypes, such as furnishing or more complex patterns.

**Table 2.** Genes identified by ViT embedding.

Feature	Symbol	Position/region	Function	Association
1	*PKHD1L1-OXR1-RSPO2-EIF3E*, *ANGPT1*, *TRHR*	chr13: 7172478–10274073	Multiple functions	Hair length and body development
	*SEMA3C*, *FGF4*	chr18: 20738192–48871585	Encodes secreted glycoprotein and fibroblast growth factor 4	Distraction index and dog hair ridge (*50*)
	*KRT71*	chr27: 44111797–44111797	Hair follicle sheath protein (*103*)	Curled hair (*48*, *49*)
	*CFAP299-FGF5*	chr32: 34869545–35498419	Cilia- and flagella-associated protein, fibroblast growth factor	Hair length
2	*PKHD1L1-OXR1-RSPO2-EIF3E*, *ANGPT1*, *TRHR*	chr13: 7295774–10212788	Multiple functions	Hair length and body development
	*HPCAL1*	chr17: 7330296–7344381	Calcium homeostasis	Conformation traits
	*PKP2*	chr27: 30491607–30491607	Proper desmosome assembly (*104*)	Chronic inflammatory skin disease (*104*)
3	*RSPO2*	chr13: 8827015–8839016	Enhances Wnt signaling	Hair length
	*HPCAL1*	chr17: 7260956–7260956	Calcium homeostasis	Conformation traits
	*MITF*	chr20: 22023329–22079634	Regulates melanocyte development	Spotting patterns in dogs
4	*CBD103*	chr16: 55463826–55468934	Antibiotic peptide	Hair color
5	*RHOT1*	chr9: 40735168–40735168	Mitochondrial transfer (*105*)	Skeletal traits (*106*)
	*RALY*-*ASIP*	chr24: 23684293–23900417	Multiple functions	Saddle tan, black-and-tan phenotypes and black coat
	*CBD103*	chr16: 55473955–55473955	Antibiotic peptide	Hair color
	*PRKAG1*	chr27: 41095397–41095397	Regulatory subunit of the AMP-activated protein kinase (*107*)	Fatness traits and weight gain (*55*, *108*)
8	*MC5R*	chr1: 24541931–24541931	Melanocortin 5 receptor	Pigmentation, immunology (*109*) and fur shedding (*56*)
	*RALY*	chr24: 23683008–23776832	Pre-mRNA splicing	Saddle tan and black-and-tan phenotypes
	*FGF5*	chr32: 35494936–35494936	Fibroblast growth factor	Hair length
15	*MC5R*	chr1: 24541931–24541931	Melanocortin 5 receptor	Pigmentation, immunology and fur shedding
	*ADRB1*	chr28: 25202379–25203119	The adrenergic receptors (*110*)	Double-layered hair and hair density (*54*)
	*CFAP299-FGF5*	chr32: 34974588–35494936	Cilia- and flagella-associated protein, fibroblast growth factor	Hair length
25	*ANGPT1*, *RSPO2*	chr13: 8431776–8952301	Multiple functions	Hair length and body development
	*IGSF3*	chr17: 54556154–54602128	Immunoglobulin-like membrane protein (*111*)	Craniofacial morphology in humans (*112*)
	*CFAP299-FGF5*	chr32: 34876069–35498419	Cilia- and flagella-associated protein, fibroblast growth factor	Hair length
28	*MITF*	chr20: 21993775–22139163	Regulates melanocyte development	Spotting patterns in dogs

*MITF* has been implicated in diverse white spotting phenotypes across several mammalian species (*47*, *52*, *53*). In dogs, the morphological variation of white spots is significant, and these phenotypes represent a continuum: solid, Irish spotting, Piebald spotting, and extreme white. In the GWAS results for R12 and V28 (Fig. 3B), several intron variants of the *MITF* gene were mapped by comparing breeds completely lacking the mutation with breeds in which it is fixed, and by integrating overlapping signals from multiple GWAS analyses that have clear main peaks in the *MITF* gene, the variant in CFA20: 22064179 strongly co-occurs with the white spotting phenotype according to the comparison (table S3).

Most of the genes identified from the ResNet-derived features were also detected in the ViT results. However, ViT pinpointed additional genes with a more precise regulatory relationship to phenotypic variation. Two variants (V15) were located upstream of the adrenoceptor β1 (*ADRB1*) gene, which has previously been associated with hair density (*54*), although no overlapping sites were found in the present study. The protein kinase AMP (adenosine 5′-monophosphate)–activated noncatalytic subunit γ1 (*PRKAG1*) gene was identified (V5), which is consistent with its known association with human weight gain (*55*). More missense variants were detected in the ViT-derived features. Notably, the variant at CFA32: 35494497, corresponding to *FGF5*, appeared in multiple feature signals (V1, V9, V20, V25, V26, and V27). Another missense mutation, Thr^237^→Ala (T237A), in the melanocortin 5 receptor (*MC5R*) (CFA1), was detected in several ViT features (V2, V8, V15, and V17); this mutation alters the protein’s tertiary structure and is strongly associated with fur shedding (*56*). Mutation in the growth hormone receptor (*GHR*) gene at CFA4: 67960816 (c.530C>T), resulting in a Pro^177^→Leu (P177L) substitution, was consistently found in small-bodied breeds such as Affenpinscher [allele frequency (AF) = 1.00], Brussels Griffon (AF = 1.00), Cavalier King Charles Spaniel (AF = 1.00), and Japanese Chin (AF = 0.67) (table S3), which is consistent with previous findings (Table 3) (*57*). More diversified genes in the ViT results verify its higher spatial resolution than that of ResNet, a superiority that likely stems from the use of a dedicated CLS (classification) token in ViT instead of global average pooling, allowing spatial representations to remain distinct.

**Table 3.** Missense variants identified from the GWAS.

Features	Symbol/Ensembl ID	Position	Protein position	Amino acid	Codons	*P* value
R1, R6, V1, V9, V20, V25, V26, V27	*FGF5*	chr32: 35494497	95	C/F	tGc/tTc	4.99 × 10^−85^
R4	*ASIP*	chr24: 23906214	138	R/C	Cgc/Tgc	5.53 × 10^−49^
V1	*PKHD1L1*	chr13: 10274073	3558	A/V	gCc/gTc	4.71 × 10^−48^
V2, V8, V15, V17	*MC5R*	chr1: 24541931	237	T/A	Acc/Gcc	1.39 × 10^−73^
V3	ENSCAFG00805012900	chr16: 36584314	1083	R/H	cGc/cAc	1.29 × 10^−43^
V7	*LIMD1*	chr20: 43273576	225	A/G	gCc/gGc	5.10 × 10^−81^
V20	*GHR*	chr4: 67960816 (*73*)	177, 184, 155, 177	P/L	cCa/cTa	1.18 × 10^−103^
V26	ENSCAFG00805019292	chr10: 26881969	134	R/H	cGc/cAc	8.82 × 10^−36^

### Uncovering endophenotypic genes via an image-derived GWAS

Our method demonstrates the ability to detect genetic variations that may have only subtle effects on phenotype but still manifest in observable traits—particularly when these differences can be captured through imaging. In our study, we identified several genes associated with hair traits in dogs, some of which had not been previously reported in this species but are known to regulate similar traits in other mammals. Notably, transcription factor 25 (*TCF25*), a gene known for its potential role in determining black stripe in sheep (*58*) and goats (*59*)—species where hair traits are often under economic selection—was also identified (V7). While many key genes affecting hair color in dogs have already been localized and validated, *TCF25* remains a likely candidate gene involved in canine hair pigmentation. There is a linked gene cluster on chromosome 24 (CFA24) in R4, which spans 1.98 Mb and contains *RALY*, eukaryotic translation initiation factor 2 subunit β (*EIF2S2*), Agouti signaling protein (*ASIP*), itchy E3 ubiquitin protein ligase (*ITCH*), phosphatidylinositol glycan anchor biosynthesis class U (*PIGU*), light chain roadblock-type 1 (*DYNLRB1*)*,* myosin heavy chain 7B (*MYH7B*), and endoplasmic reticulum degradation–enhancing α-mannosidase–like protein 2 (*EDEM2*), among others. *EIF2S2* has been implicated in pigmentation regulation across diverse species, including birds, mammals, and humans, suggesting its conserved role in controlling coloration traits (*60*–*62*). In addition, *ITCH*, *MYH7B*, and *EDEM2* are associated with melanoma and pigmentation phenotypes (*63**–**65*). There is a facial stripe in goats called “facciuto” that is associated with *DYNLRB1* and *PIGU*, which were reported to be involved in the coat color of alpacas. The presence of multiple pigmentation-related genes within this CFA24 region suggests that these genes may act in concert to regulate pigment deposition and color pattern formation in dogs. Thyrotropin-releasing hormone receptor (*TRHR*) is believed to affect weight gain (*66*, *67*) and to regulate hair growth through thyrotropin-releasing hormone–mediated pathways in human hair follicles (*68*). Consistent with these findings, in our study, *TRHR* was identified within the hair-related signaling clusters in the V1 and R1 features, supporting its potential role as a candidate gene involved in regulating hair growth in dogs.

Missense variants often cause protein structure alterations, potentially affecting the protein function. We collected missense variants from 45 GWAS annotation results. In addition to variants in *FGF5* and *MC5R*, a variant was detected in *ASIP*, which encodes an antagonist of *MC1R*, a receptor that plays a role in regulating the switch between eumelanin and pheomelanin production in melanocytes (*69*). The *ASIP* Arg^96^→Cys (R96C) missense mutation (c.427C>T, exon 4) causes a recessive black coat color in dogs by disrupting the ASIP function and leading to increased eumelanin production (*70*). In our analysis, a variant (c.412C>T, exon 5) leading to R96C also showed a significant association with the endophenotype (R4). Given the sequence similarity and positional alignment of this region, our results indicate that this mutation is the same as the previously described *ASIP* R96C (c.427C>T) substitution. The minor AF at this locus is ~0.067 in the Dog10K dataset; its mutation results in the replacement of phaeomelanin with eumelanin but does not affect the white spotting pattern controlled by *MITF*. In the dog breeds with a high frequency of mutation, this mutation leads to darker coat breeds: Mudi (AF = 0.9), Small Munsterlander (AF = 0.86), Puli (AF = 0.75), Affenpinscher (AF = 0.7), Schipperke (AF = 0.67), and Giant Schnauzer (AF = 0.58), while the distribution of white markings remains unchanged (Newfoundland: 0.67; American Eskimo Dog: 0.58) (table S3).

A variant was identified at chr16: 36584314 (c.3248G>A), resulting in an amino acid substitution, Arg^1083^→His (R1083H), within the gene ENSCAFG00805012900 (V3). The corresponding canine transcript (ENSCAFT00805023539.1) encodes a protein that is strongly orthologous to lipoprotein receptor–related protein 5–like (*LRP5L*) (*e* value = 0; score = 2575.2). Given the structural similarity between *LRP5L* and *LRP5*, this gene may participate in related biological processes such as lipid metabolism and bone formation (*71*, *72*). This variant occurs in only a few breeds, with the highest AF observed in Clumber Spaniel (AF = 0.90), which is characterized by a heavy bone structure and high body weight. A variant at chr10: 26881969 (c.401G>A) resulting in an Arg^134^→His (R134H) substitution in the homolog of apolipoprotein B mRNA editing catalytic polypeptide–like 3H (*APOBEC3H*) (seed ortholog: 9668; ENSMPUP00000013141; *e* value = 3.56 × 10^−84^; score = 261) (Table 3) has not yet been determined to influence canine appearance.

## DISCUSSION

In summary, our study demonstrates the power of using deep learning–based feature embeddings for high-throughput phenotyping and GWAS in dogs. We built an image dataset of 181 breeds containing 13,254 images. By leveraging trained classification deep neural networks—ResNet and ViT—we extract high-dimensional image features that reflect rich visual traits of canine morphology—without the need for manual phenotypic annotations. These embeddings serve as a form of parameter-free dimensionality reduction that reduces the need for manual annotation and captures latent phenotypic structures—including subtle morphological traits that are not readily observable or classifiable by humans. These unsupervised visual features, derived from UMAP embeddings, can be used as quantitative phenotypes in GWASs to identify morphology-associated genes. By using this method to convert visual differences in dog breeds into quantitative endophenotypes, we mapped 15 previously reported genes associated with dog phenotypes and more loci with potential roles in shaping dog morphological traits.

Because R1 and V1 are strongly correlated with coat length and the major hair length gene *FGF5* on CFA32, they clearly capture the primary differences in hair type and length among breeds. R2 and V2 also map to CFA13 (*OXR1*, *RSPO2*, *EIF3E*, etc.) but are not significantly associated with coat length (R2: *r* = 0.02, *P* = 0.82; V2: *r* = 0.037, *P* = 0.66), suggesting that they capture more complex local hair traits beyond hair length, such as moustache and eyebrow growth (*OXR1*) (*5*). These results indicate that additional genes contribute to the traits represented by R2 and V2, including ENSCAFG00805006704, a gene encoding long noncoding RNA (R2 and V2) in CFA17, and *RCL1* (V2) in CFA1, which is associated with tail curl (*73*). A cluster of variants on CFA18 (20.55 to 20.89 Mb) exhibited strong linkage within breeds and was repeatedly identified across multiple GWAS analyses (R1, R4, V1, and V27). Most of these variants are located in intronic or intergenic regions, whereas a few are positioned downstream, upstream, or within the 3′ untranslated regions of cluster of differentiation 36 (*CD36*) and semaphorin 3C (*SEMA3C*). Taking the highly significant variant CFA18: 20817004 as an example, the high-mutation breeds are also small-body-size dwarf dogs (Biewer Terrier, Cairn Terrier, Cardigan Welsh Corgi, Coton de Tulear, Glen of Imaal Terrier, Norfolk Terrier, etc.). We also identified a variant, CFA18: 48871585, located in the 3′ untranslated region of *FGF4*, a gene associated with a short-leg phenotype (*74*), and it is correlated with CFA18: 20817004 interbreeds (*r* = −0.88, *P* < 0.001) (table S3), which is consistent with the possibility that long-range LD further affects fat metabolism and body weight (*75*, *76*).

Unlike previous traditional dog GWASs, endophenotypes were derived through a two-step feature extraction and low-dimensional embedding process rather than relying on individual measurement values or breed-level averages of traits such as height, coat length, and body size (*4*, *10*). On the basis of the feature extraction capability of classification models and structure preserving of UMAP (*77*), endophenotypes with rich information can be derived by embedding high-dimensional image features into a lower-dimensional manifold that retains both local and global similarity structures among samples, thereby ensuring consistent phenotypic representations within breeds. We not only identified the widely reported coat-determining genes *FGF5*, *RSPO2*, *KRT71*, *ASIP*, and *CBD103* and their key mutations in dogs (*5*, *39*, *56*) but also detected *TCF25*, a gene that appears in multiple livestock and poultry GWAS results for hair and plumage color (*58*, *59*, *78*, *79*), suggesting its conserved function and potential role in coat color variation in dogs. Unlike approaches that directly use model embeddings or apply PCA for dimensionality reduction (*24*, *80*, *81*), we adopted the nonlinear UMAP method. The strong local structure preservation of UMAP helps highlight biologically meaningful variations, especially in the first few features related to coat length, coat color, and body size. By combining the representational power of deep learning with UMAP’s ability to maintain both local and global structures, our method generates compact and informative features that enhance downstream biological analyses.

These features often capture nonlinear, composite relationships that can be highly predictive for classification or clustering but are difficult to disentangle into individual, interpretable traits. Consequently, when UMAP embeddings are used as quantitative phenotypes in GWASs, interpreting significant genetic associations in terms of specific phenotypes becomes challenging. UMAP does not ensure that each embedding dimension corresponds to a distinct or independent biological attribute, and while arranging feature values and contrasting extreme breeds can sometimes reveal patterns, this approach is less effective for composite phenotypes where multiple traits are intertwined within a single feature. As a result, significant SNP-feature associations may reflect complex, composite signals rather than individual traits, limiting their utility for biological interpretation or validation. To further clarify the nature of these embeddings, PCA of genome-wide SNPs revealed a clear population structure across breeds (fig. S8), whereas Mantel tests showed low correlations between genotype- and image-based embeddings (ResNet: *r* = 0.043, *P* = 0.066; ViT: *r* = 0.088, *P* = 0.073). These results indicate that the learned visual embeddings primarily capture morphological variation rather than the overall genetic distance. Because UMAP embeddings are nonlinear projections rather than direct biological measurements, any detected associations capture statistical correlations rather than causal relationships, and inferring causality between genotypes and endophenotypes remains difficult.

There is substantial research potential in the identification of novel genes for the breeding of dogs and other economically valuable species. A large number of genes associated with phenotypic traits have been identified, some of which have been previously reported in dogs, whereas others have been shown to be related to hair growth, body development, and other body traits. In our study, the embedding features were derived from whole-body images of dogs. Future work could use semantic segmentation and object detection techniques to analyze individual body parts and integrate contrastive learning to enhance feature extraction for GWASs (*24*, *82*, *83*). Such approaches may help overcome the limitations of information loss and reduce the influence of noise arising from unrelated image regions.

## MATERIALS AND METHODS

### Genomic data screening and image dataset construction

We downloaded the VCF files of the SNPs and their indices from Dog10k. Genotype data in VCF format were processed using PLINK version 1.9. Variants with a minor AF < 0.01 or an extremely high missing mutation rate (>99.9%) were excluded, and biallelic SNPs were retained for downstream analyses. To avoid sampling bias and interference from nonbreed dogs, we removed sample information for breeds with fewer than five individuals or nonbreed dogs. Following quality control filtering, we retained 839 individuals from 128 breeds and 33,210,250 variants (data S1), resulting in an overall genotyping rate of 0.9967.

Owing to the limited breed coverage and high background noise in existing public dog image datasets, we constructed a custom image dataset. Raw images were downloaded using an automated script based on the Image-Downloader framework (https://github.com/HeroPPPPath/Image-Downloader-master) by querying the Bing image search engine with standardized dog breed names as keywords. To maximize breed diversity, a broad range of dog breeds was queried. All the images were subsequently subjected to a manual curation process by two independent reviewers. Each image was categorized as retained, discarded, or retained after processing according to predefined criteria, including breed consistency, exclusion of puppies, absence of severe occlusion, sufficient image quality, and minimal human-body interference. Images labeled as retained after processing underwent cropping to remove human interference. Only images independently approved by both reviewers (retained or retained after processing) were retained for downstream analysis, while images marked as discarded by either reviewer were excluded. On the basis of the availability of corresponding genomic data, 181 dog breeds were ultimately retained, resulting in a final dataset of 13,254 high-quality images.

### Feature extraction

We used two feature extractor architectures, ResNet50 (ResNet; a residual network) and ViT-B/16 (ViT; a vision transformer), for their strong representational capabilities across visual domains (*84*). Both models were pretrained on the ImageNet dataset (*85*), and we trained them on a supervised classification task comprising 181 dog breeds to learn general morphological features. Model training was optimized using the cross-entropy loss function with the Adam optimizer. The networks were subsequently fine-tuned for embedding generation. For ResNet50, the original fully connected and softmax layers were removed, and a fully connected layer with a specified output dimension was inserted after the average pooling layer in layer 4. The resulting embeddings were saved in HDF5 format. Similarly, for ViT-B/16, the classification head was replaced by a fully connected layer that mapped the class token to the same specified dimensional space. In subsequent analyses, both models produced 256-dimensional embeddings.

### Grad-CAM analysis

We trained a SegFormer model (*86*) on the Oxford-IIIT Pet Dataset (*87*), splitting 80% of the data for training and 20% for validation, to generate segmentation masks of our dataset with three classes (dog, contour, and background) using cross-entropy loss and the AdamW optimizer, achieving a high mean Intersection over Union on the held-out validation set. To assess whether the models focused on biologically relevant image regions, we applied Grad-CAM using the pytorch_grad_cam library (*88*, *89*). Heatmaps were generated by computing gradients with respect to the predicted class at the architecturally relevant final feature layer for each image (the last residual block for ResNet and the last layer normalization for ViT). The resulting activation maps were upsampled to match the input resolution and aligned with the segmentation masks to separate the dog and background regions. Activation values between the two regions were compared using a one-sided Mann-Whitney *U* test (*P* < 0.001 considered significant), and an area under the curve–based effect size was calculated to estimate the probability that activations in dog regions exceeded those in background regions.

### Correlation analysis

Pearson correlation analysis was performed to assess both the consistency of feature representations across models and the biological relevance of embedding features (see data S2 to S4 for breed-averaged embedding features and morphological data). All correlation coefficients (*r*) and *P* values were obtained using the cor.test() function in R (version 4.4.2), with the parameter method = “Pearson.” Missing values were removed using complete.cases().

### Region-level masking with superpixel perturbation

To further validate the robustness and biological relevance of the learned visual features, we performed region-level masking on the basis of semantic segmentation by the SLIC superpixel algorithm using the slic function from the scikit-image library. The process partitions each image into 200 compact superpixels while keeping the background pixels unchanged. The compactness parameters were set to 40, 60, and 80 to control the level of texture detail retained within each superpixel. This results in progressively larger and more uniform regions that increasingly suppress fine-grained fur texture information. For each compactness setting, features were first extracted using the ResNet and ViT models and subsequently reduced to the same dimensionality as the original operation (ResNet: 13 features; ViT: 32 features) using UMAP, enabling a direct comparison across different perturbation levels.

### Feature dimensionality reduction

During dimensionality reduction, we applied t-SNE for visualization and UMAP to project the high-dimensional image embeddings into a lower-dimensional space. For t-SNE, we used the implementation from scikit-learn (sklearn.manifold. TSNE) with parameters random_state = 42, early_exaggeration = 4, and perplexity = 45. To quantitatively assess whether samples of the same breed cluster together, we calculated the intra/interclass distance ratio, defined as the mean pairwise distance between samples within a breed divided by the mean distance to samples of other breeds (*90*). A smaller ratio indicates tighter within-breed clustering relative to between-breed separation, reflecting the higher discriminability of the embedded space. For UMAP, we applied the umap-learn package (umap.UMAP) with the following parameters: n_neighbors = 5, min_dist = 0.05, and metric = “cosine.”

We adopted a dynamic strategy for determining the number of dimensions to retain. Instead of setting a fixed number of output dimensions, we used a variance-based stopping criterion: We continued to increase the dimensionality until three consecutive dimensions exhibited a variance contribution of less than 1%. This threshold ensures that the retained dimensions carry meaningful structural information from the original embedding space while eliminating redundant or noise-dominated dimensions. The features from ResNet50 were reduced to 13 features to perform the GWAS. However, we reduced the number of features from ViT to 32 because ViT can extract more abundant details.

The initialization for UMAP is critical. There are two commonly used methods for UMAP initialization—LE and random—and LE initialization yields better results in multiple datasets (*77*). The advantage of LE initialization arises from its ability to provide a globally consistent low-dimensional representation that respects the local manifold structure from the start of optimization. The goal is to find a low-dimensional representation Y to satisfy the following

$$\text{min}\sum_{i,j}{\left\|y_{i}-y_{j}\right\|}^{2}W_{\mathrm{ij}}$$ (1)

This problem can be reformulated in quadratic form as follows

$$\text{min}\mathrm{tr}\left(Y^{T}\mathrm{LY}\right),\;\text{subject to}\;Y^{T}\mathrm{DY}=I$$ (2)

where L=D−W is the graph Laplacian. The solution is given by the eigenvectors for the smallest eigenvalues of Ly=λDy (*91*). From spectral graph theory, we know that for a connected graph, the first eigenvalue of the Laplacian is always zero and that its corresponding eigenvector is constant across all nodes, which is uninformative for embedding. The subsequent eigenvectors—associated with the smallest nonzero eigenvalues—vary slowly over the graph and correspond to smooth functions on the vertices. As the eigenvalue increases, the corresponding eigenvectors oscillate more rapidly between adjacent nodes. Therefore, the lower-frequency (smoother) eigenvectors capture the broad, slowly varying structures of the data that reflect their global manifold geometry. Feature smoothness was proposed as a metric for quantifying information in graph data (*92*).

### Construction of embedded phenotypic trees

A hierarchical clustering approach was applied to construct the phylogenetic tree. Pairwise Euclidean distances between samples were first computed using the dist() function in R, and the resulting distance matrix was then subjected to agglomerative hierarchical clustering with the average linkage method [UPGMA (unweighted pair group method with arithmetic mean)] implemented in the hclust() function. Associations between the phenotypic tree structure and morphological traits were assessed using PERMANOVA (permutational analysis of variance; adonis2 function in the vegan R package). Continuous traits, including coat length, body weight, height, weight-to-height ratio, EV, and CI, were standardized before analysis. The ear type (erect versus lop) was treated as a categorical factor. For each trait, a distance matrix of the phenotypic tree was used as the response variable, and the statistical significance was estimated using 999 permutations. For multivariate analysis, multiple traits were included simultaneously in the model to evaluate their combined contribution to the phenotypic tree structure.

### Genome-wide association study

In dog GWASs, breed-level phenotypic representations are typically used to characterize heritable traits at the breed scale (*4*, *93*). After dimensionality reduction using UMAP, the embeddings from images of the same breed formed compact clusters because of their shared visual phenotype. To maintain the stability of the breed-level features, we removed outlier images within each breed cluster using *z*-score filtering, with a threshold of 2. The remaining embeddings were treated as a representative phenotypic feature set for each breed. To generate individual-level phenotypes for GWAS, phenotypic feature vectors were randomly assigned from the corresponding breed-level feature set to individual dogs on the basis of the breed labels in the genotype data. Original genotype data in VCF format were converted into the PLINK binary format (BED, BIM, and FAM) using PLINK version 1.9 (*94*), incorporating the generated phenotype files and allowing for nonstandard chromosomes. To account for the population structure and genetic relatedness, a kinship matrix was computed using GEMMA (Genome-wide Efficient Mixed Model Association; version 0.98) (*95*).

GWAS was performed separately for each phenotypic feature using linear mixed models in GEMMA. Both the kinship matrix and association tests were based on 10,460,815 SNPs, thereby controlling for the population structure and cryptic relatedness. To assess potential inflation or deflation of test statistics arising from population stratification or limited statistical power, the λ_GC_ (genomic inflation factor) was calculated for each GWAS. Only results with λ_GC_ values between 0.95 and 1.05 were retained for downstream analyses to ensure well-calibrated association statistics. To identify statistically independent genome-wide significant signals while accounting for LD, we performed LD clumping using PLINK. This procedure retained the most significant SNP within a 500-kb window, removing other SNPs with an LD *r*^2^ > 0.8. Genome-wide significance was defined by a Bonferroni-corrected *P* value threshold of 4.78 × 10^−9^ (α = 0.05/10,460,815 tested variants). The resulting set of independent, genome-wide significant variants was then extracted from the original VCF using BCFtools (*96*).

### Gene annotation

We extracted the clumped SNPs from the GWAS results and performed variant annotation using the Ensembl VEP (Variant Effect Predictor; version 112.0) (*97*). The annotation was conducted using the German Shepherd (UU_Cfam_GSD_1.0) breed-specific reference genome. Gene symbols and variant functional consequences were annotated on the basis of the Ensembl database (data S5 and S6).

### Functional annotation of the uncharacterized genes

The protein sequence encoded by the uncharacterized gene was searched against eggNOG (version 5.0) (http://eggnog.embl.de/) (*98*). Default search parameters were applied. Functional annotations were obtained from the corresponding orthologous group, including Clusters of Orthologous Groups functional categories, Kyoto Encyclopedia of Genes and Genomes pathways, and Pfam protein domains. Closely related orthologous proteins from multiple taxa were identified on the basis of sequence similarity.

### Model training environment

All the above deep learning models were implemented in PyTorch and trained on a Windows-based system equipped with NVIDIA graphics processing units (GPUs). Experiments were conducted using Python 3.12 and PyTorch 2.6.0, with CUDA 11.8 and cuDNN enabled for GPU acceleration. All the experiments were performed on an NVIDIA RTX 4090 D GPU.

### Genotype embeddings and comparison with image features

To generate genotype embeddings, we performed PCA on genome-wide SNP genotypes and retained the top 50 principal components, which reflect the population structure and genetic relationships among breeds. To evaluate the correspondence between genotype and image-derived embeddings, we computed pairwise Euclidean distance matrices for the top principal components (genotype embeddings) and for the ResNet- or ViT-based image embeddings (breed averaged). We then performed Mantel tests using Spearman correlation with 999 permutations to assess the association between the genotype and image distances.

## References

[1] G. D. Wang, W. Zhai, H. C. Yang, R. X. Fan, X. Cao, L. Zhong, L. Wang, F. Liu, H. Wu, L. G. Cheng, A. D. Poyarkov, N. A. Poyarkov Jr., S. S. Tang, W. M. Zhao, Y. Gao, X. M. Lv, D. M. Irwin, P. Savolainen, C. I. Wu, Y. P. Zhang, The genomics of selection in dogs and the parallel evolution between dogs and humans. Nat. Commun. 4, 1860 (2013). 23673645 10.1038/ncomms2814

[2] S. J. Zhang, L. Scarsbrook, H. Li, A. Carmagnini, S. Charlton, T. Feuerborn, G. Boeskorov, G. Chen, J. M. Deom, E. A. Dimopoulos, K. Dobney, J. Dong, L. Du, A. J. Hansen, A. Harris, G. Hernández-Alonso, X. Jia, A. Kim, G. M. Li, R. Li, A. Linderholm, A. Outram, M. Qiu, L. Ren, Q. Ruan, R. Sala, A. Stepanov, Y. Sun, K. Tabbada, O. Thalmann, V. Varfolomeev, L. Wang, Q. Wang, S. Wang, W. Wei, Y. Yang, J. Yin, V. Zaibert, Z. Zhang, G. Dong, E. Rosengren, M. S. Sinding, E. A. Ostrander, G. Larson, M. Ma, L. A. F. Frantz, G. D. Wang, Genomic evidence for the Holocene codispersal of dogs and humans across Eastern Eurasia. Science 390, 735–740 (2025). 41231995 10.1126/science.adu2836

[3] American Kennel Club, *The Complete Dog Book: 20th Edition* (Random House Publishing Group, 2007).

[4] J. Plassais, J. Kim, B. W. Davis, D. M. Karyadi, A. N. Hogan, A. C. Harris, B. Decker, H. G. Parker, E. A. Ostrander, Whole genome sequencing of canids reveals genomic regions under selection and variants influencing morphology. Nat. Commun. 10, 1489 (2019). 30940804 10.1038/s41467-019-09373-w PMC6445083

[5] E. Cadieu, M. W. Neff, P. Quignon, K. Walsh, K. Chase, H. G. Parker, B. M. Vonholdt, A. Rhue, A. Boyko, A. Byers, A. Wong, D. S. Mosher, A. G. Elkahloun, T. C. Spady, C. André, K. G. Lark, M. Cargill, C. D. Bustamante, R. K. Wayne, E. A. Ostrander, Coat variation in the domestic dog is governed by variants in three genes. Science 326, 150–153 (2009). 19713490 10.1126/science.1177808 PMC2897713

[6] M. Oguro-Okano, M. Honda, K. Yamazaki, K. Okano, Mutations in the melanocortin 1 receptor, β-defensin103 and agouti signaling protein genes, and their association with coat color phenotypes in Akita-inu dogs. J. Vet. Med. Sci. 73, 853–858 (2011). 21321476 10.1292/jvms.10-0439

[7] M. Kang, B. Ahn, S. Youk, Y. M. Lee, J. J. Kim, J. H. Ha, C. Park, Tracing the origin of the RSPO2 long-hair allele and epistatic interaction between FGF5 and RSPO2 in sapsaree dog. Genes (Basel) 13, 102 (2022). 35052442 10.3390/genes13010102 PMC8775186

[8] E. A. Ostrander, F. H. Epstein, Lecture., Both ends of the leash—The human links to good dogs with bad genes. N. Engl. J. Med. 367, 636–646 (2012). 22894576 10.1056/NEJMra1204453 PMC3508784

[9] A. L. Shearin, E. A. Ostrander, Leading the way: Canine models of genomics and disease. Dis. Model. Mech. 3, 27–34 (2010). 20075379 10.1242/dmm.004358 PMC4068608

[10] Y. Momozawa, A. C. Merveille, G. Battaille, M. Wiberg, J. Koch, J. L. Willesen, H. F. Proschowsky, V. Gouni, V. Chetboul, L. Tiret, M. Fredholm, E. H. Seppälä, H. Lohi, M. Georges, A. S. Lequarré, Genome wide association study of 40 clinical measurements in eight dog breeds. Sci. Rep. 10, 6520 (2020). 32300138 10.1038/s41598-020-63457-y PMC7162946

[11] Y. Momozawa, The potential of translational research in dogs in human medicine. Transl. Reg. Sci. 1, 31–36 (2019).

[12] E. Axelsson, A. Ratnakumar, M. L. Arendt, K. Maqbool, M. T. Webster, M. Perloski, O. Liberg, J. M. Arnemo, A. Hedhammar, K. Lindblad-Toh, The genomic signature of dog domestication reveals adaptation to a starch-rich diet. Nature 495, 360–364 (2013). 23354050 10.1038/nature11837

[13] X. Wang, L. Zhu, Y. Lan, G. Li, T. Zhou, Q. Huang, T. Yuan, C. Tian, Z. Fan, Z. Zhang, G.-D. Wang, Convergent evolution of metabolic functions: Evidence from the gut microbiomes of humans and dogs. iMetaOmics 3, e70059 (2026).

[14] H. Brian, W. Victoria, W. Richard, The self-domestication hypothesis: Evolution of bonobo psychology is due to selection against aggression. Anim. Behav. 83, 573–585 (2012).

[15] R. M. Buckley, E. A. Ostrander, Large-scale genomic analysis of the domestic dog informs biological discovery. Genome Res. 34, 811–821 (2024). 38955465 10.1101/gr.278569.123 PMC11293549

[16] Y. Li, B. M. Vonholdt, A. Reynolds, A. R. Boyko, R. K. Wayne, D. D. Wu, Y. P. Zhang, Artificial selection on brain-expressed genes during the domestication of dog. Mol. Biol. Evol. 30, 1867–1876 (2013). 23660689 10.1093/molbev/mst088

[17] B. M. Vonholdt, J. P. Pollinger, K. E. Lohmueller, E. Han, H. G. Parker, P. Quignon, J. D. Degenhardt, A. R. Boyko, D. A. Earl, A. Auton, A. Reynolds, K. Bryc, A. Brisbin, J. C. Knowles, D. S. Mosher, T. C. Spady, A. Elkahloun, E. Geffen, M. Pilot, W. Jedrzejewski, C. Greco, E. Randi, D. Bannasch, A. Wilton, J. Shearman, M. Musiani, M. Cargill, P. G. Jones, Z. Qian, W. Huang, Z. L. Ding, Y. P. Zhang, C. D. Bustamante, E. A. Ostrander, J. Novembre, R. K. Wayne, Genome-wide SNP and haplotype analyses reveal a rich history underlying dog domestication. Nature 464, 898–902 (2010). 20237475 10.1038/nature08837 PMC3494089

[18] N. Moravčíková, R. Kasarda, R. Židek, L. Vostrý, H. Vostrá-Vydrová, J. Vašek, D. Čílová, Czechoslovakian wolfdog genomic divergence from its ancestors *Canis lupus*, german shepherd dog, and different sheepdogs of European origin. Genes (Basel) 12, 832 (2021). 34071464 10.3390/genes12060832 PMC8228135

[19] M. S. Sinding, S. Gopalakrishnan, J. Ramos-Madrigal, M. de Manuel, V. V. Pitulko, L. Kuderna, T. R. Feuerborn, L. A. F. Frantz, F. G. Vieira, J. Niemann, J. A. Samaniego Castruita, C. Carøe, E. U. Andersen-Ranberg, P. D. Jordan, E. Y. Pavlova, P. A. Nikolskiy, A. K. Kasparov, V. V. Ivanova, E. Willerslev, P. Skoglund, M. Fredholm, S. E. Wennerberg, M. P. Heide-Jørgensen, R. Dietz, C. Sonne, M. Meldgaard, L. Dalén, G. Larson, B. Petersen, T. Sicheritz-Pontén, L. Bachmann, Ø. Wiig, T. Marques-Bonet, A. J. Hansen, M. T. P. Gilbert, Arctic-adapted dogs emerged at the Pleistocene-Holocene transition. Science 368, 1495–1499 (2020). 32587022 10.1126/science.aaz8599 PMC7116267

[20] S. J. Zhang, J. Ma, M. Riera, S. Besenbacher, J. E. Niskanen, N. Salokorpi, S. Hundi, M. K. Hytönen, T. Zhou, G. M. Li, E. A. Ostrander, M. H. Schierup, H. Lohi, G. D. Wang, Determinants of de novo mutations in extended pedigrees of 43 dog breeds. Genome Biol. 26, 305 (2025). 40999484 10.1186/s13059-025-03804-2 PMC12465969

[21] N. B. Sutter, M. A. Eberle, H. G. Parker, B. J. Pullar, E. F. Kirkness, L. Kruglyak, E. A. Ostrander, Extensive and breed-specific linkage disequilibrium in *Canis familiaris*. Genome Res. 14, 2388–2396 (2004). 15545498 10.1101/gr.3147604 PMC534662

[22] C. Sommer, D. W. Gerlich, Machine learning in cell biology—Teaching computers to recognize phenotypes. J. Cell Sci. 126, 5529–5539 (2013). 24259662 10.1242/jcs.123604

[23] J. P. Pirruccello, M. D. Chaffin, E. L. Chou, S. J. Fleming, H. Lin, M. Nekoui, S. Khurshid, S. F. Friedman, A. G. Bick, A. Arduini, L. C. Weng, S. H. Choi, A. D. Akkad, P. Batra, N. R. Tucker, A. W. Hall, C. Roselli, E. J. Benjamin, S. K. Vellarikkal, R. M. Gupta, C. M. Stegmann, D. Juric, J. R. Stone, R. S. Vasan, J. E. Ho, U. Hoffmann, S. A. Lubitz, A. A. Philippakis, M. E. Lindsay, P. T. Ellinor, Deep learning enables genetic analysis of the human thoracic aorta. Nat. Genet. 54, 40–51 (2022). 34837083 10.1038/s41588-021-00962-4 PMC8758523

[24] Z. Xie, T. Zhang, S. Kim, J. Lu, W. Zhang, C. H. Lin, M. R. Wu, A. Davis, R. Channa, L. Giancardo, H. Chen, S. Wang, R. Chen, D. Zhi, iGWAS: Image-based genome-wide association of self-supervised deep phenotyping of retina fundus images. PLOS Genet. 20, e1011273 (2024). 38728357 10.1371/journal.pgen.1011273 PMC11111076

[25] S. R. Islam, Z. Xie, W. He, D. Zhi, Vision transformer autoencoders for unsupervised representation learning: Capturing local and non-local features in brain imaging to reveal genetic associations. medRxiv [Preprint] (2025); 10.1101/2025.03.24.25324549. PMC13635212 42265418

[26] K. He, X. Zhang, S. Ren, J. Sun, “Deep residual learning for image recognition,” in *Proceedings of the IEEE Conference on Computer Vision and Pattern Recognition* (IEEE, 2016), pp. 770–778.

[27] A. Dosovitskiy, L. Beyer, A. Kolesnikov, D. Weissenborn, X. Zhai, T. Unterthiner, M. Dehghani, M. Minderer, G. Heigold, S. Gelly, “An image is worth 16x16 words: Transformers for image recognition at scale,” in *International Conference on Learning Representations 2021* (ICLR, 2021).

[28] L. McInnes, J. Healy, UMAP: Uniform manifold approximation and projection for dimension reduction. arXiv:1802.03426 [stat.ML] (2018).

[29] Z. Liu, R. Ma, Y. Zhong, Assessing and improving reliability of neighbor embedding methods: A map-continuity perspective. Nat. Commun. 16, 5037 (2025). 40447630 10.1038/s41467-025-60434-9 PMC12125374

[30] M. Raghu, T. Unterthiner, S. Kornblith, C. Zhang, A. Dosovitskiy, “Do vision transformers see like convolutional neural networks?” in *Proceedings of the 35th International Conference on Neural Information Processing Systems* (Curran Associates Inc., 2021), pp. 12116–12128.

[31] K. Won Hee, K. Sangil, C. Alix, C. Je-Yoel, S. Seunggwan, Genome-wide statistical evidence elucidates candidate factors of life expectancy in dogs. Mol. Cells 48, 100162 (2025). 39580055 10.1016/j.mocell.2024.100162 PMC11721540

[32] A. M. Balcarcel, M. R. Sánchez-Villagra, A. Evin, M. Nussbaumer, A. Hemelsdaël, M. Geiger, Breed function and behaviour correlate with endocranial volume in domestic dogs. Biol. Lett. 20, 20240342 (2024). 39532143 10.1098/rsbl.2024.0342 PMC11557248

[33] C. Dierks, S. Mömke, U. Philipp, O. Distl, Allelic heterogeneity of FGF5 mutations causes the long-hair phenotype in dogs. Anim. Genet. 44, 425–431 (2013). 23384345 10.1111/age.12010

[34] C. Drögemüller, S. Rüfenacht, B. Wichert, T. Leeb, Mutations within the FGF5 gene are associated with hair length in cats. Anim. Genet. 38, 218–221 (2007). 17433015 10.1111/j.1365-2052.2007.01590.x

[35] R. Legrand, L. Tiret, M. Abitbol, Two recessive mutations in FGF5 are associated with the long-hair phenotype in donkeys. Genet. Sel. Evol. 46, 65 (2014). 25927731 10.1186/s12711-014-0065-5 PMC4175617

[36] G. Li, S. Zhou, C. Li, B. Cai, H. Yu, B. Ma, Y. Huang, Y. Ding, Y. Liu, Q. Ding, C. He, J. Zhou, Y. Wang, G. Zhou, Y. Li, Y. Yan, J. Hua, B. Petersen, Y. Jiang, T. Sonstegard, X. Huang, Y. Chen, X. Wang, Base pair editing in goat: Nonsense codon introgression into FGF5 results in longer hair. FEBS J. 286, 4675–4692 (2019). 31276295 10.1111/febs.14983

[37] M. A. Haque, N. K. Kim, R. Yeji, B. Lee, J. H. Ha, Y. M. Lee, J. J. Kim, Genomic prediction and genome-wide association studies of morphological traits and distraction index in Korean Sapsaree dogs. PLOS ONE 19, e0312583 (2024). 39570887 10.1371/journal.pone.0312583 PMC11581321

[38] Q. Wu, X. Han, Y. Zhang, H. Liu, H. Zhou, K. Wang, J. Han, One copy number variation within the angiopoietin-1 gene is associated with Leizhou black goat meat quality. Animals 14, 2682 (2024). 39335271 10.3390/ani14182682 PMC11428527

[39] N. Ninausz, P. Fehér, E. Csányi, M. Heltai, L. Szabó, E. Barta, P. Kemenszky, G. Sándor, F. Jánoska, M. Horváth, S. Kusza, K. Frank, L. Varga, V. Stéger, White and other fur colourations and hybridization in golden jackals (*Canis aureus*) in the Carpathian basin. Sci. Rep. 13, 21969 (2023). 38082037 10.1038/s41598-023-49265-0 PMC10713657

[40] M. Guo, Z. Zhao, R. Wang, X. Zheng, Y. Peng, Z. Liu, X. Li, Y. Gong, Activity and transcriptional regulatory elements of the promoter in Arctic fox (*Vulpes lagopus*) β-defensin103 gene. Sheng Wu Gong Cheng Xue Bao 35, 1469–1477 (2019). 31441618 10.13345/j.cjb.190063

[41] S. I. Candille, C. B. Kaelin, B. M. Cattanach, B. Yu, D. A. Thompson, M. A. Nix, J. A. Kerns, S. M. Schmutz, G. L. Millhauser, G. S. Barsh, A β-defensin mutation causes black coat color in domestic dogs. Science 318, 1418–1423 (2007). 17947548 10.1126/science.1147880 PMC2906624

[42] C. Chen, C. Liu, X. Xiong, S. Fang, H. Yang, Z. Zhang, J. Ren, Y. Guo, L. Huang, Copy number variation in the MSRB3 gene enlarges porcine ear size through a mechanism involving miR-584-5p. Genet. Sel. Evol. 50, 72 (2018). 30587124 10.1186/s12711-018-0442-6 PMC6307293

[43] J. M. Paris, A. Letko, I. M. Häfliger, P. Ammann, C. Drögemüller, Ear type in sheep is associated with the MSRB3 locus. Anim. Genet. 51, 968–972 (2020). 32805068 10.1111/age.12994

[44] M. T. Webster, N. Kamgari, M. Perloski, M. P. Hoeppner, E. Axelsson, Å. Hedhammar, G. Pielberg, K. Lindblad-Toh, Linked genetic variants on chromosome 10 control ear morphology and body mass among dog breeds. BMC Genomics 16, 474 (2015). 26100605 10.1186/s12864-015-1702-2 PMC4477608

[45] X. Cao, C. Ling, Y. Liu, Y. Gu, J. Huang, W. Sun, Pleiotropic gene HMGA2 regulates myoblast proliferation and affects body size of sheep. Animals 14, 2721 (2024). 39335310 10.3390/ani14182721 PMC11428621

[46] D. L. Dreger, H. G. Parker, E. A. Ostrander, S. M. Schmutz, Identification of a mutation that is associated with the saddle tan and black-and-tan phenotypes in Basset Hounds and Pembroke Welsh Corgis. J. Hered. 104, 399–406 (2013). 23519866 10.1093/jhered/est012

[47] I. Baranowska Körberg, E. Sundström, J. R. Meadows, G. Rosengren Pielberg, U. Gustafson, Å. Hedhammar, E. K. Karlsson, J. Seddon, A. Söderberg, C. Vilà, X. Zhang, M. Åkesson, K. Lindblad-Toh, G. Andersson, L. Andersson, A simple repeat polymorphism in the MITF-M promoter is a key regulator of white spotting in dogs. PLOS ONE 9, e104363 (2014). 25116146 10.1371/journal.pone.0104363 PMC4130573

[48] T. Kuramoto, R. Hirano, M. Kuwamura, T. Serikawa, Identification of the rat Rex mutation as a 7-bp deletion at splicing acceptor site of the Krt71 gene. J. Vet. Med. Sci. 72, 909–912 (2010). 20179389 10.1292/jvms.09-0554

[49] B. Gandolfi, C. A. Outerbridge, L. G. Beresford, J. A. Myers, M. Pimentel, H. Alhaddad, J. C. Grahn, R. A. Grahn, L. A. Lyons, The naked truth: Sphynx and Devon Rex cat breed mutations in KRT71. Mamm. Genome 21, 509–515 (2010). 20953787 10.1007/s00335-010-9290-6 PMC2974189

[50] N. H. Salmon Hillbertz, M. Isaksson, E. K. Karlsson, E. Hellmén, G. R. Pielberg, P. Savolainen, C. M. Wade, H. von Euler, U. Gustafson, A. Hedhammar, M. Nilsson, K. Lindblad-Toh, L. Andersson, G. Andersson, Duplication of FGF3, FGF4, FGF19 and ORAOV1 causes hair ridge and predisposition to dermoid sinus in Ridgeback dogs. Nat. Genet. 39, 1318–1320 (2007). 17906623 10.1038/ng.2007.4

[51] E. A. Brown, P. J. Dickinson, T. Mansour, B. K. Sturges, M. Aguilar, A. E. Young, C. Korff, J. Lind, C. L. Ettinger, S. Varon, R. Pollard, C. T. Brown, T. Raudsepp, D. L. Bannasch, FGF4 retrogene on CFA12 is responsible for chondrodystrophy and intervertebral disc disease in dogs. Proc. Natl. Acad. Sci. U.S.A. 114, 11476–11481 (2017). 29073074 10.1073/pnas.1709082114 PMC5664524

[52] S. Hofstetter, F. Seefried, I. M. Häfliger, V. Jagannathan, T. Leeb, C. Drögemüller, A non-coding regulatory variant in the 5′-region of the MITF gene is associated with white-spotted coat in Brown Swiss cattle. Anim. Genet. 50, 27–32 (2019). 30506810 10.1111/age.12751

[53] J. L. Petersen, R. L. Sieck, D. J. Steffen, White coat color of a Black Angus calf attributed to an occurrence of the delR217 variant of MITF. Anim. Genet. 54, 549–552 (2023). 37062854 10.1111/age.13327

[54] D. T. Whitaker, E. A. Ostrander, Hair of the dog: Identification of a cis-regulatory module predicted to influence canine coat composition. Genes (Basel) 10, 323 (2019). 31035530 10.3390/genes10050323 PMC6562840

[55] G. Jassim, J. Fernø, F. M. Theisen, M. Haberhausen, A. Christoforou, B. Håvik, S. Gebhardt, H. Remschmidt, C. Mehler-Wex, J. Hebebrand, S. Lehellard, V. M. Steen, Association study of energy homeostasis genes and antipsychotic-induced weight gain in patients with schizophrenia. Pharmacopsychiatry 44, 15–20 (2011). 20821366 10.1055/s-0030-1263174

[56] J. J. Hayward, M. G. Castelhano, K. C. Oliveira, E. Corey, C. Balkman, T. L. Baxter, M. L. Casal, S. A. Center, M. Fang, S. J. Garrison, S. E. Kalla, P. Korniliev, M. I. Kotlikoff, N. S. Moise, L. M. Shannon, K. W. Simpson, N. B. Sutter, R. J. Todhunter, A. R. Boyko, Complex disease and phenotype mapping in the domestic dog. Nat. Commun. 7, 10460 (2016). 26795439 10.1038/ncomms10460 PMC4735900

[57] M. Rimbault, H. C. Beale, J. J. Schoenebeck, B. C. Hoopes, J. J. Allen, P. Kilroy-Glynn, R. K. Wayne, N. B. Sutter, E. A. Ostrander, Derived variants at six genes explain nearly half of size reduction in dog breeds. Genome Res. 23, 1985–1995 (2013). 24026177 10.1101/gr.157339.113 PMC3847769

[58] L. Ma, W. Zhao, Q. Ma, J. Wang, Z. Zhao, J. Zhang, Y. Gu, Genome-wide association study of birth wool length, birth weight, and head color in Chinese tan sheep through whole-genome re-sequencing. Animals 14, 3495 (2024). 39682459 10.3390/ani14233495 PMC11640532

[59] X. Sun, Q. Niu, J. Jiang, G. Wang, P. Zhou, J. Li, C. Chen, L. Liu, L. Xu, H. Ren, Identifying candidate genes for litter size and three morphological traits in Youzhou dark goats based on genome-wide SNP markers. Genes (Basel) 14, 1183 (2023). 37372363 10.3390/genes14061183 PMC10298679

[60] S. Rashid, I. Molotkov, N. Klebanov, M. Shaughnessy, M. J. Daly, M. Artomov, H. Tsao, Mendelian randomization analysis reveals inverse genetic risks between skin cancers and vitiligo. JID Innov. 3, 100217 (2023). 38034848 10.1016/j.xjidi.2023.100217 PMC10685305

[61] J. Guo, H. Tao, P. Li, L. Li, T. Zhong, L. Wang, J. Ma, X. Chen, T. Song, H. Zhang, Whole-genome sequencing reveals selection signatures associated with important traits in six goat breeds. Sci. Rep. 8, 10405 (2018). 29991772 10.1038/s41598-018-28719-w PMC6039503

[62] S. Wang, S. Rohwer, D. R. de Zwaan, D. P. L. Toews, I. J. Lovette, J. Mackenzie, D. Irwin, Selection on a small genomic region underpins differentiation in multiple color traits between two warbler species. Evol. Lett. 4, 502–515 (2020). 33312686 10.1002/evl3.198 PMC7719548

[63] I. Stefanaki, O. A. Panagiotou, E. Kodela, H. Gogas, K. P. Kypreou, F. Chatzinasiou, V. Nikolaou, M. Plaka, I. Kalfa, C. Antoniou, J. P. Ioannidis, E. Evangelou, A. J. Stratigos, Replication and predictive value of SNPs associated with melanoma and pigmentation traits in a Southern European case-control study. PLOS ONE 8, e55712 (2013). 23393597 10.1371/journal.pone.0055712 PMC3564929

[64] G. N. Chirițoiu, M. Chirițoiu, C. V. A. Munteanu, Dataset of human EDEM2 melanoma cells proteomics, affinity proteomics and deglycoproteomics. Data Brief 39, 107471 (2021). 34712749 10.1016/j.dib.2021.107471 PMC8529096

[65] F. Lévy, K. Muehlethaler, S. Salvi, A. L. Peitrequin, C. K. Lindholm, J. C. Cerottini, D. Rimoldi, Ubiquitylation of a melanosomal protein by HECT-E3 ligases serves as sorting signal for lysosomal degradation. Mol. Biol. Cell 16, 1777–1787 (2005). 15703212 10.1091/mbc.E04-09-0803 PMC1073660

[66] X. G. Liu, L. J. Tan, S. F. Lei, Y. J. Liu, H. Shen, L. Wang, H. Yan, Y. F. Guo, D. H. Xiong, X. D. Chen, F. Pan, T. L. Yang, Y. P. Zhang, Y. Guo, N. L. Tang, X. Z. Zhu, H. Y. Deng, S. Levy, R. R. Recker, C. J. Papasian, H. W. Deng, Genome-wide association and replication studies identified TRHR as an important gene for lean body mass. Am. J. Hum. Genet. 84, 418–423 (2009). 19268274 10.1016/j.ajhg.2009.02.004 PMC2668008

[67] X. Jiang, Z. Cai, X. Zhao, L. Zhang, Z. Chen, Y. Wang, X. Guo, N. Xu, Mapping, cDNA cloning and tissue expression of the porcine thyrotropin-releasing hormone receptor gene. Anim. Biotechnol. 22, 30–36 (2011). 21328103 10.1080/10495398.2011.547745

[68] E. Gáspár, C. Hardenbicker, E. Bodó, B. Wenzel, Y. Ramot, W. Funk, A. Kromminga, R. Paus, Thyrotropin releasing hormone (TRH): A new player in human hair-growth control. FASEB J. 24, 393–403 (2010). 19825978 10.1096/fj.08-126417

[69] D. L. Bannasch, C. B. Kaelin, A. Letko, R. Loechel, P. Hug, V. Jagannathan, J. Henkel, P. Roosje, M. K. Hytönen, H. Lohi, M. Arumilli, K. M. Minor, J. R. Mickelson, C. Drögemüller, G. S. Barsh, T. Leeb, Dog colour patterns explained by modular promoters of ancient canid origin. Nat. Ecol. Evol. 5, 1415–1423 (2021). 34385618 10.1038/s41559-021-01524-x PMC8484016

[70] J. A. Kerns, J. Newton, T. G. Berryere, E. M. Rubin, J. F. Cheng, S. M. Schmutz, G. S. Barsh, Characterization of the dog Agouti gene and a nonagoutimutation in German Shepherd Dogs. Mamm. Genome 15, 798–808 (2004). 15520882 10.1007/s00335-004-2377-1

[71] D. Li, J. Zhu, M. Zhang, Q. Shi, R. Guo, D. Zhang, P. Zheng, H. Zhang, G. Li, J. Wu, G. Sun, Q. Wen, J. Tan, Z. Liu, X. Liu, H. Yang, H. Lu, G. Cao, Z. Yin, Q. Wang, SOSTDC1 downregulation in CD4^+^ T cells confers protection against obesity-induced insulin resistance. Cell Rep. 44, 115496 (2025). 40173040 10.1016/j.celrep.2025.115496

[72] B. O. Williams, LRP5: From bedside to bench to bone. Bone 102, 26–30 (2017). 28341377 10.1016/j.bone.2017.03.044

[73] A. Vaysse, A. Ratnakumar, T. Derrien, E. Axelsson, G. Rosengren Pielberg, S. Sigurdsson, T. Fall, E. H. Seppälä, M. S. Hansen, C. T. Lawley, E. K. Karlsson, D. Bannasch, C. Vilà, H. Lohi, F. Galibert, M. Fredholm, J. Häggström, A. Hedhammar, C. André, K. Lindblad-Toh, C. Hitte, M. T. Webster, Identification of genomic regions associated with phenotypic variation between dog breeds using selection mapping. PLOS Genet. 7, e1002316 (2011). 22022279 10.1371/journal.pgen.1002316 PMC3192833

[74] H. G. Parker, B. M. VonHoldt, P. Quignon, E. H. Margulies, S. Shao, D. S. Mosher, T. C. Spady, A. Elkahloun, M. Cargill, P. G. Jones, C. L. Maslen, G. M. Acland, N. B. Sutter, K. Kuroki, C. D. Bustamante, R. K. Wayne, E. A. Ostrander, An expressed fgf4 retrogene is associated with breed-defining chondrodysplasia in domestic dogs. Science 325, 995–998 (2009). 19608863 10.1126/science.1173275 PMC2748762

[75] S. Verpoorten, P. Sfyri, D. Scully, R. Mitchell, A. Tzimou, V. Mougios, K. Patel, A. Matsakas, Loss of CD36 protects against diet-induced obesity but results in impaired muscle stem cell function, delayed muscle regeneration and hepatic steatosis. Acta Physiol (Oxf.) 228, e13395 (2020). 31599493 10.1111/apha.13395

[76] N. Mejhert, F. Wilfling, D. Esteve, J. Galitzky, V. Pellegrinelli, C. I. Kolditz, N. Viguerie, J. Tordjman, E. Näslund, P. Trayhurn, D. Lacasa, I. Dahlman, V. Stich, P. Lång, D. Langin, A. Bouloumié, K. Clément, M. Rydén, Semaphorin 3C is a novel adipokine linked to extracellular matrix composition. Diabetologia 56, 1792–1801 (2013). 23666167 10.1007/s00125-013-2931-z

[77] D. Kobak, G. C. Linderman, Initialization is critical for preserving global data structure in both t-SNE and UMAP. Nat. Biotechnol. 39, 156–157 (2021). 33526945 10.1038/s41587-020-00809-z

[78] S. Peng, L. Zhang, S. Ali, S. Lin, Z. Liao, Q. Peng, P. Xie, Z. Zhang, Whole genome resequencing uncovers candidate genes related to plumage color in Yuexi frizzled feather chicken. Poult. Sci. 104, 105680 (2025). 40840286 10.1016/j.psj.2025.105680 PMC12396410

[79] H. Liu, Y. Xi, Q. Tang, J. Qi, Z. Zhou, Z. Guo, W. Fan, J. Hu, Y. Xu, S. Liang, M. Xie, J. Tang, Y. Zhang, S. Hou, Genetic fine-mapping reveals single nucleotide polymorphism mutations in the MC1R regulatory region associated with duck melanism. Mol. Ecol. 32, 3076–3088 (2023). 36929535 10.1111/mec.16924

[80] M. Kirchler, S. Konigorski, M. Norden, C. Meltendorf, M. Kloft, C. Schurmann, C. Lippert, transferGWAS: GWAS of images using deep transfer learning. Bioinformatics 38, 3621–3628 (2022). 35640976 10.1093/bioinformatics/btac369

[81] K. Patel, Z. Xie, H. Yuan, S. M. S. Islam, Y. Xie, W. He, W. Zhang, A. Gottlieb, H. Chen, L. Giancardo, A. Knaack, E. Fletcher, M. Fornage, S. Ji, D. Zhi, Unsupervised deep representation learning enables phenotype discovery for genetic association studies of brain imaging. Commun. Biol. 7, 414 (2024). 38580839 10.1038/s42003-024-06096-7 PMC10997628

[82] T. Zhou, L. Li, X. Li, C. M. Feng, J. Li, L. Shao, Group-wise learning for weakly supervised semantic segmentation. IEEE Trans. Image Process. 31, 799–811 (2022). 34910633 10.1109/TIP.2021.3132834

[83] P. Wang, X. Shen, Z. Lin, S. Cohen, B. Price, A. Yuille, “Joint object and part segmentation using deep learned potentials,” in *Proceedings of the 2015 IEEE International Conference on Computer Vision (ICCV)* (IEEE Computer Society, 2015), pp. 1573–1581.

[84] H. Yan, V. Mubonanyikuzo, T. E. Komolafe, L. Zhou, T. Wu, N. Wang, Hybrid-RViT: Hybridizing ResNet-50 and vision transformer for enhanced Alzheimer's disease detection. PLOS ONE 20, e0318998 (2025). 39951414 10.1371/journal.pone.0318998 PMC11828341

[85] J. Deng, W. Dong, R. Socher, L.-J. Li, K. Li, L. Fei-Fei, “ImageNet: A large-scale hierarchical image database,” in *2009 IEEE Conference on Computer Vision and Pattern Recognition* (IEEE, 2009), pp. 248–255.

[86] E. Xie, W. Wang, Z. Yu, A. Anandkumar, J. M. Alvarez, P. Luo, “SegFormer: Simple and efficient design for semantic segmentation with transformers,” in *Proceedings of the 35th International Conference on Neural Information Processing Systems* (Curran Associates Inc., 2021), pp. 12077–12090.

[87] O. M. Parkhi, A. Vedaldi, A. Zisserman, C. V. Jawahar, “Cats and dogs,” in *2012 IEEE Conference on Computer Vision and Pattern Recognition* (IEEE, 2012), pp. 3498–3505.

[88] F.-G. Fernandez, TorchCAM: class activation explorer, version 1.2.0, GitHub (2020); https://github.com/frgfm/torch-cam.

[89] J. Gildenblat, Contributors, PyTorch library for CAM methods, version 1.1.0, GitHub (2021); https://github.com/jacobgil/pytorch-grad-cam.

[90] T. Shoubiao, L. Li, P. Chunyu, S. Ling, Image-to-class distance ratio: A feature filtering metric for image classification. Neurocomputing 165, 211–221 (2015).

[91] M. Belkin, P. Niyogi, Laplacian eigenmaps for dimensionality reduction and data representation. Neural Comput. 15, 1373–1396 (2003).

[92] Y. Hou, J. Zhang, J. Cheng, K. Ma, R. T. B. Ma, H. Chen, M.-C. Yang, “Measuring and improving the use of graph information in graph neural networks,” in *International Conference on Learning Representations 2020* (ICLR, 2020).

[93] P. Jones, K. Chase, A. Martin, P. Davern, E. A. Ostrander, K. G. Lark, Single-nucleotide-polymorphism-based association mapping of dog stereotypes. Genetics 179, 1033–1044 (2008). 18505865 10.1534/genetics.108.087866 PMC2429857

[94] C. C. Chang, C. C. Chow, L. C. Tellier, S. Vattikuti, S. M. Purcell, J. J. Lee, Second-generation PLINK: Rising to the challenge of larger and richer datasets. Gigascience 4, 7 (2015). 25722852 10.1186/s13742-015-0047-8 PMC4342193

[95] X. Zhou, M. Stephens, Genome-wide efficient mixed-model analysis for association studies. Nat. Genet. 44, 821–824 (2012). 22706312 10.1038/ng.2310 PMC3386377

[96] P. Danecek, J. K. Bonfield, J. Liddle, J. Marshall, V. Ohan, M. O. Pollard, A. Whitwham, T. Keane, S. A. McCarthy, R. M. Davies, H. Li, Twelve years of SAMtools and BCFtools. Gigascience 10, giab008 (2021). 33590861 10.1093/gigascience/giab008 PMC7931819

[97] W. McLaren, L. Gil, S. E. Hunt, H. S. Riat, G. R. Ritchie, A. Thormann, P. Flicek, F. Cunningham, The Ensembl variant effect predictor. Genome Biol. 17, 122 (2016). 27268795 10.1186/s13059-016-0974-4 PMC4893825

[98] J. Huerta-Cepas, D. Szklarczyk, D. Heller, A. Hernández-Plaza, S. K. Forslund, H. Cook, D. R. Mende, I. Letunic, T. Rattei, L. J. Jensen, C. von Mering, P. Bork, eggNOG 5.0: A hierarchical, functionally and phylogenetically annotated orthology resource based on 5090 organisms and 2502 viruses. Nucleic Acids Res. 47, D309–D314 (2018). 10.1093/nar/gky1085 PMC6324079 30418610

[99] S. Andrino, V. Lorenzo, S. Dunner, E. Contreras, J. Cañón, N. Sevane, Syringohydromyelia in dogs: The genomic component underlying a complex neurological disease. Animals 12, 2622 (2022). 36230363 10.3390/ani12192622 PMC9558965

[100] D. Rajawat, S. S. Nayak, K. Jain, A. Sharma, S. Parida, S. P. Sahoo, B. Bhushan, D. B. Patil, T. Dutt, M. Panigrahi, Genomic patterns of selection in morphometric traits across diverse Indian cattle breeds. Mamm. Genome 35, 377–389 (2024). 39014170 10.1007/s00335-024-10047-2

[101] M. E. MacDonald, C. M. Ambrose, M. P. Duyao, R. H. Myers, C. Lin, L. Srinidhi, G. Barnes, S. A. Taylor, M. James, N. Groot, H. MacFarlane, B. Jenkins, M. A. Anderson, N. S. Wexler, J. F. Gusella, G. P. Bates, S. Baxendale, H. Hummerich, S. Kirby, M. North, S. Youngman, R. Mott, G. Zehetner, Z. Sedlacek, A. Poustka, A.-M. Frischauf, H. Lehrach, A. J. Buckler, D. Church, L. Doucette-Stamm, M. C. O'Donovan, L. Riba-Ramirez, M. Shah, V. P. Stanton, S. A. Strobel, K. M. Draths, J. L. Wales, P. Dervan, D. E. Housman, M. Altherr, R. Shiang, L. Thompson, T. Fielder, J. J. Wasmuth, D. Tagle, J. Valdes, L. Elmer, M. Allard, L. Castilla, M. Swaroop, K. Blanchard, F. S. Collins, R. Snell, T. Holloway, K. Gillespie, N. Datson, D. Shaw, P. S. Harper, A novel gene containing a trinucleotide repeat that is expanded and unstable on Huntington's disease chromosomes. Cell 72, 971–983 (1993). 8458085 10.1016/0092-8674(93)90585-e

[102] A. R. Young, M. Narita, Oncogenic HMGA2: Short or small? Genes Dev. 21, 1005–1009 (2007). 17473167 10.1101/gad.1554707

[103] L. Langbein, M. A. Rogers, S. Praetzel-Wunder, B. Helmke, P. Schirmacher, J. Schweizer, K25 (K25irs1), K26 (K25irs2), K27 (K25irs3), and K28 (K25irs4) represent the type I inner root sheath keratins of the human hair follicle. J. Invest. Dermatol. 126, 2377–2386 (2006). 16874310 10.1038/sj.jid.5700494

[104] K. Tengvall, S. Kozyrev, M. Kierczak, K. Bergvall, F. H. Farias, B. Ardesjö-Lundgren, M. Olsson, E. Murén, R. Hagman, T. Leeb, G. Pielberg, Å. Hedhammar, G. Andersson, K. Lindblad-Toh, Multiple regulatory variants located in cell type-specific enhancers within the PKP2 locus form major risk and protective haplotypes for canine atopic dermatitis in German shepherd dogs. BMC Genet. 17, 97 (2016). 27357287 10.1186/s12863-016-0404-3 PMC4928279

[105] M. Jing, X. Xiong, X. Mao, Q. Song, L. Zhang, Y. Ouyang, Y. Pang, Y. Fu, W. Yan, HMGB1 promotes mitochondrial transfer between hepatocellular carcinoma cells through RHOT1 and RAC1 under hypoxia. Cell Death Dis. 15, 155 (2024). 38378644 10.1038/s41419-024-06536-6 PMC10879213

[106] X. Gao, S. Zhou, Z. Liu, D. Ruan, J. Wu, J. Quan, E. Zheng, J. Yang, G. Cai, Z. Wu, M. Yang, Genome-wide association study for somatic skeletal traits in Duroc × (Landrace × Yorkshire) pigs. Animals 14, 37 (2024). 10.3390/ani14010037 PMC10778498 38200769

[107] B. Faubert, E. E. Vincent, M. C. Poffenberger, R. G. Jones, The AMP-activated protein kinase (AMPK) and cancer: Many faces of a metabolic regulator. Cancer Lett. 356, 165–170 (2015). 24486219 10.1016/j.canlet.2014.01.018

[108] O. Demeure, L. Liaubet, J. Riquet, D. Milan, Determination of PRKAG1 coding sequence and mapping of PRKAG1 and PRKAG2 relatively to porcine back fat thickness QTL. Anim. Genet. 35, 123–125 (2004). 15025572 10.1111/j.1365-2052.2004.01102.x

[109] X. Zhang, J. Su, T. Huang, X. Wang, C. Wu, J. Li, J. Li, J. Zhang, Y. Wang, Characterization of the chicken melanocortin 5 receptor and its potential role in regulating hepatic glucolipid metabolism. Front. Physiol. 13, 917712 (2022). 36277187 10.3389/fphys.2022.917712 PMC9583845

[110] E. F. Kelley, E. M. Snyder, B. D. Johnson, Influence of β-1 adrenergic receptor genotype on cardiovascular response to exercise in healthy subjects. Cardiol. Res. 9, 343–349 (2018). 30627284 10.14740/cr785 PMC6306116

[111] S. Saupe, G. Roizès, M. Peter, S. Boyle, K. Gardiner, A. De Sario, Molecular cloning of a human cDNA IGSF3 encoding an immunoglobulin-like membrane protein: Expression and mapping to chromosome band 1p13. Genomics 52, 305–311 (1998). 9790749 10.1006/geno.1998.5439

[112] W. Wu, G. Zhai, Z. Xu, B. Hou, D. Liu, T. Liu, W. Liu, F. Ren, Whole-exome sequencing identified four loci influencing craniofacial morphology in northern Han Chinese. Hum. Genet. 138, 601–611 (2019). 30968251 10.1007/s00439-019-02008-6 PMC6554238

[113] T. Zhou, S. Y. Pu, S. J. Zhang, Q. J. Zhou, M. Zeng, J. S. Lu, X. Lu, Y. N. Wang, G. D. Wang, Dog10K: An integrated Dog10K database summarizing canine multi-omics. Nucleic Acids Res. 53, D939–D947 (2025). 39436034 10.1093/nar/gkae928 PMC11701641

