# Supplementary material for: Image feature embedding with a deep learning framework improves genome-wide association studies on dog endophenotypes
Source: Sci Adv. 2026 Jun 24;12(26):eaee1088. doi: 10.1126/sciadv.aee1088 (PMC13292930; doi:10.1126/sciadv.aee1088)
Supplement: Supplementary file 1 — Figs. S1 to S8 Tables S1 to S3 Legends for data S1 to S6 [file sciadv.aee1088_sm.pdf]

Science Advances
AAAS

Supplementary Materials for

Image feature embedding with a deep learning framework improves genome-wide association studies on dog endophenotypes

Guang-Xiao E and Guo-Dong Wang

Corresponding author: Guo-Dong Wang, wanggd@mail.kiz.ac.cn

Sci. Adv. **12**, eaee1088 (2026)
DOI: 10.1126/sciadv.aee1088

The PDF file includes:

Figs. S1 to S8
Tables S1 to S3
Legends for data S1 to S6

Other Supplementary Material for this manuscript includes the following:

Data S1 to S6

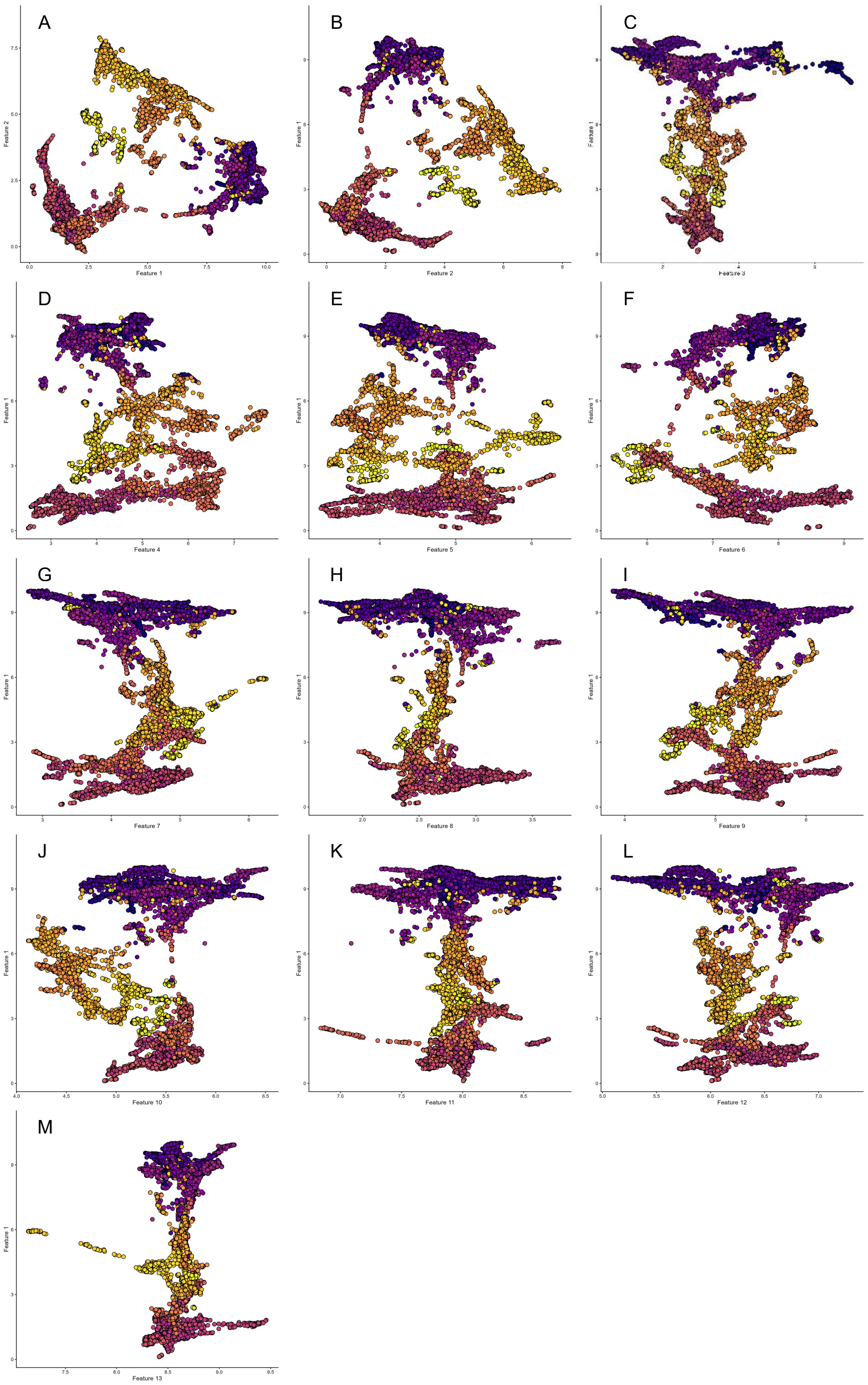

Fig. S1. UMAP scatter plots for ResNet embedding features. (**A**)–(**M**) show Features R1–R13 on the x-axis. Outliers were removed prior to plotting.

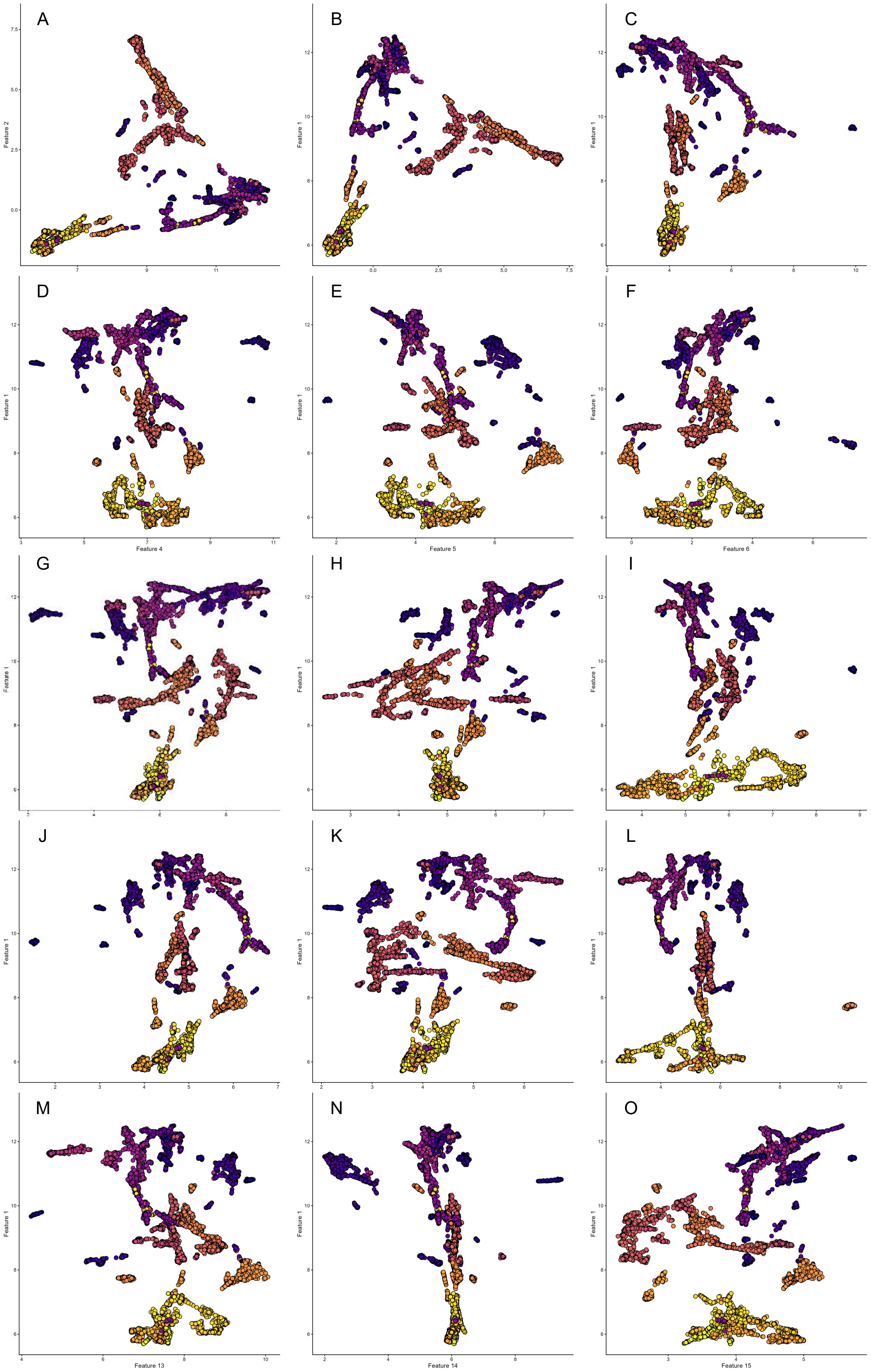

A
B
C
D
E
F
G
H
I
J
K
L
M
N
O
Feature 1
Feature 2
Feature 4
Feature 5
Feature 6
Feature 13
Feature 14
Feature 15

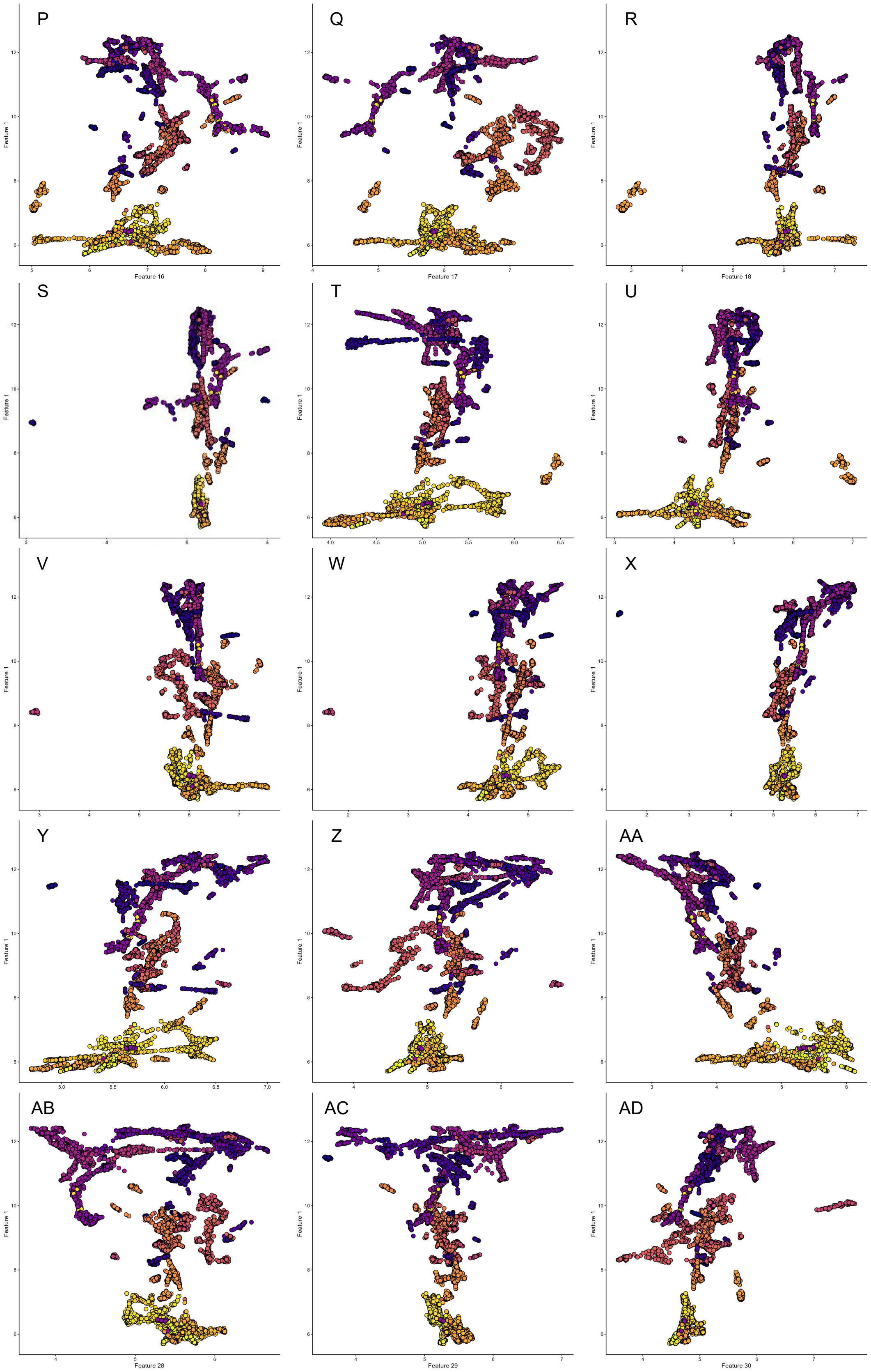

P
Q
R
S
T
U
V
W
X
Y
Z
AA
AB
AC
AD
Feature 1
Feature 16
Feature 17
Feature 18
Feature 28
Feature 29
Feature 30

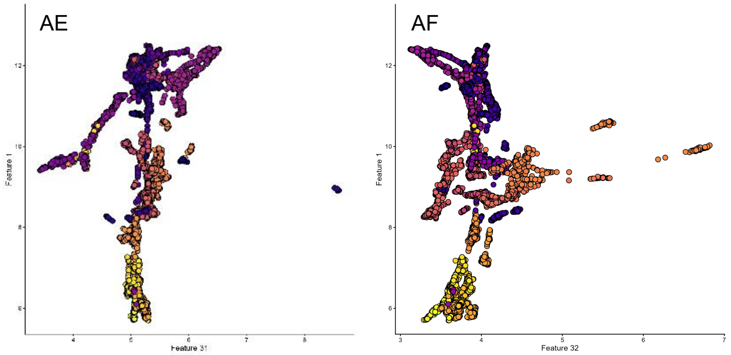

Fig. S2. UMAP scatter plots for ViT embedding features. (**A**)–(**AF**) show Features V1–V32 on the x-axis. Outliers were removed prior to plotting.

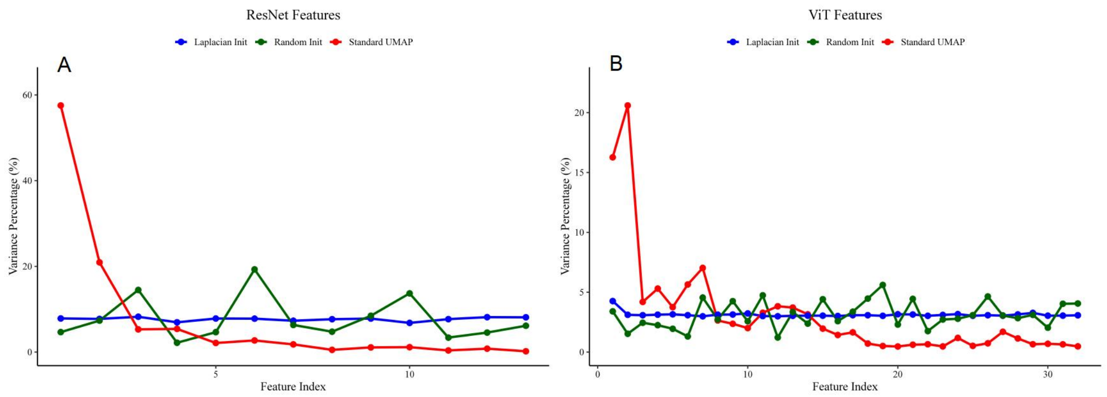

Fig. S3. Inter-breeds variance of mean features across different embedding dimensions for ResNet and ViT. Blue, green, and red lines represent Laplacian Eigenmaps (LE), randomly initialized UMAP, and LE initialized UMAP embeddings, respectively. (**A**) Inter-breeds variance of mean features across embedding dimensions for ResNet. (**B**) Inter-breeds variance of mean features across embedding dimensions for ViT.

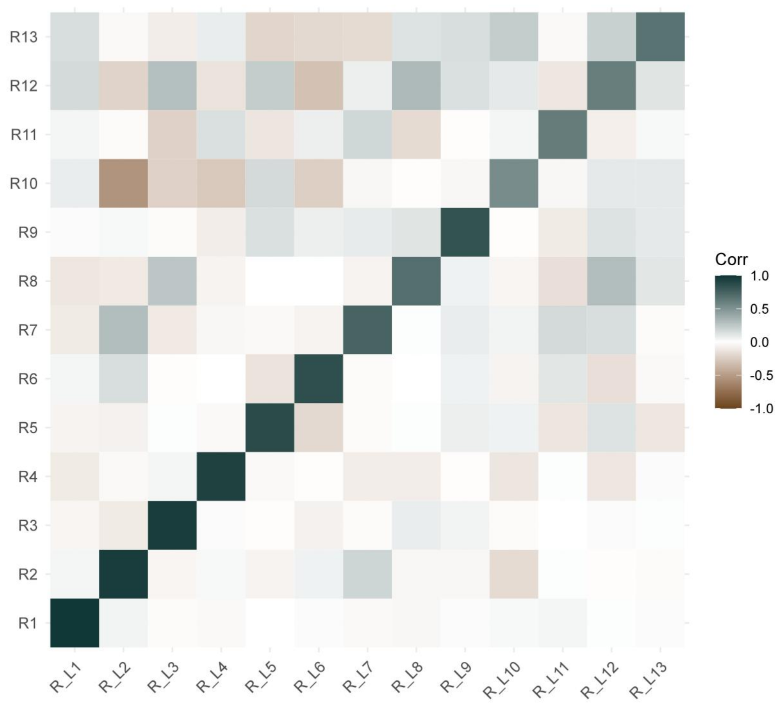

Fig. S4. Correlation heatmap of mean features across breeds between LE and LE-initialized UMAP embedding features from ResNet. Each cell represents the correlation of trait averages for breeds (LE embedding features are labeled as R_L1–R_L13, LE-initialized UMAP embedding features are labeled as R1–R13).

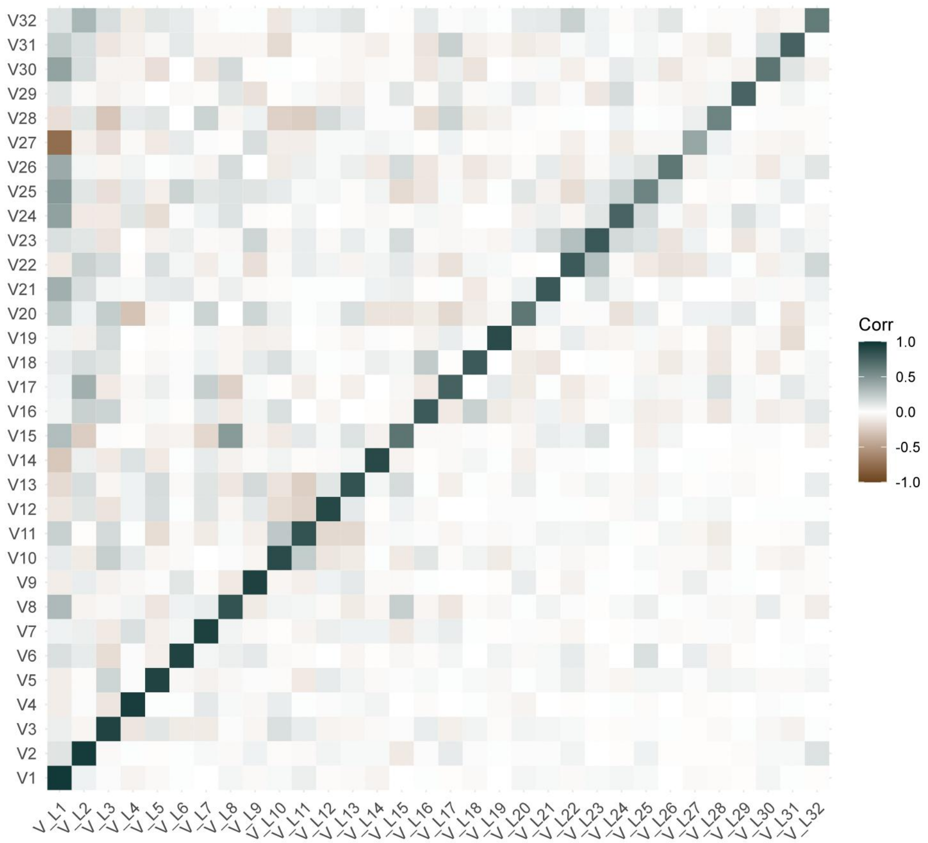

Fig. S5. Correlation heatmap of mean features across breeds between LE and LE-initialized UMAP embedding features from ViT. Each cell represents the correlation of trait averages for breeds (LE embedding features are labeled as V_L1–V_L13, LE-initialized UMAP embedding features are labeled as V1–V13).

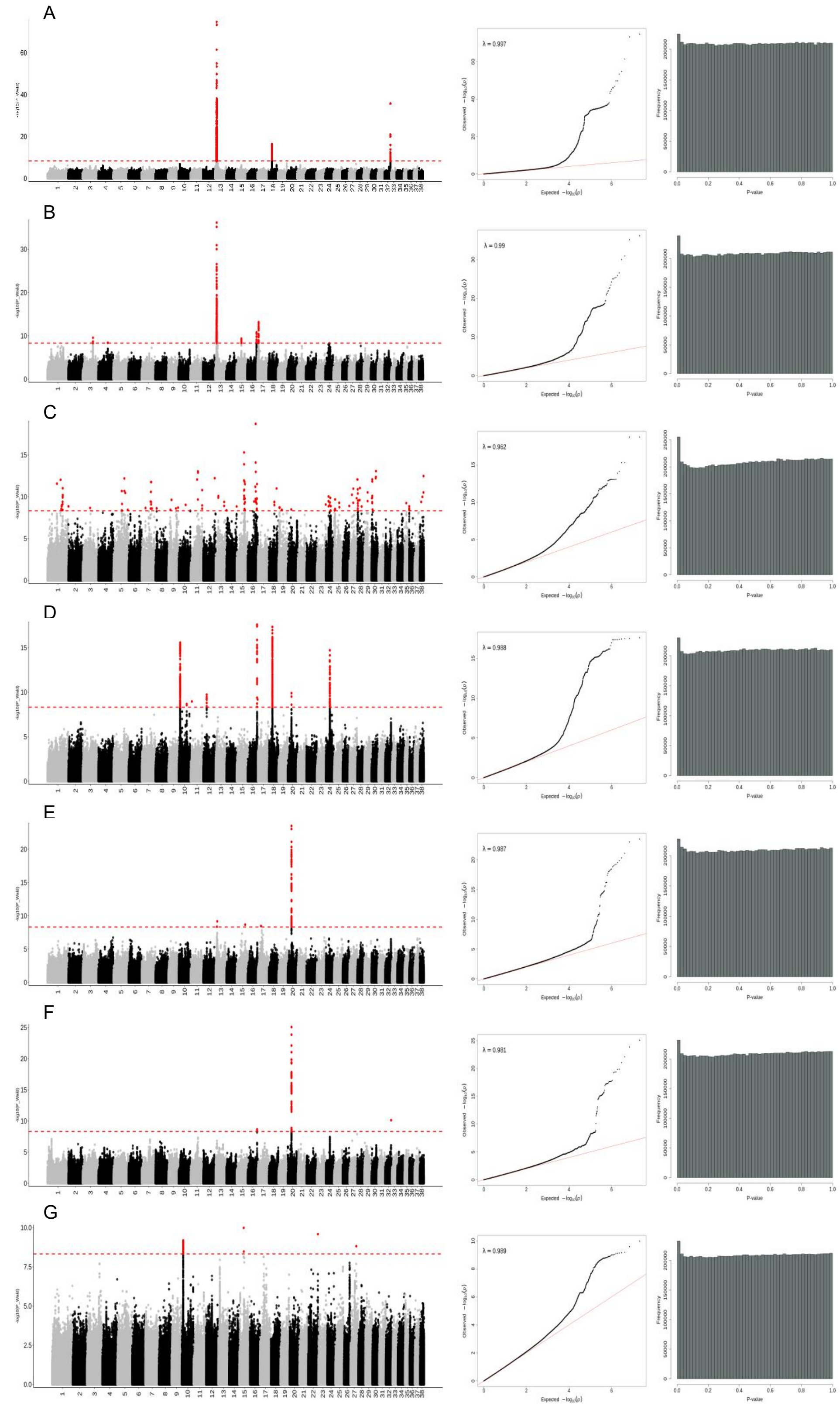
A
B
C
D
E
F
G
Expected −log10(p)
Observed −log10(p)
Frequency
P-value
λ = 0.997
λ = 0.99
λ = 0.962
λ = 0.988
λ = 0.987
λ = 0.981
λ = 0.989

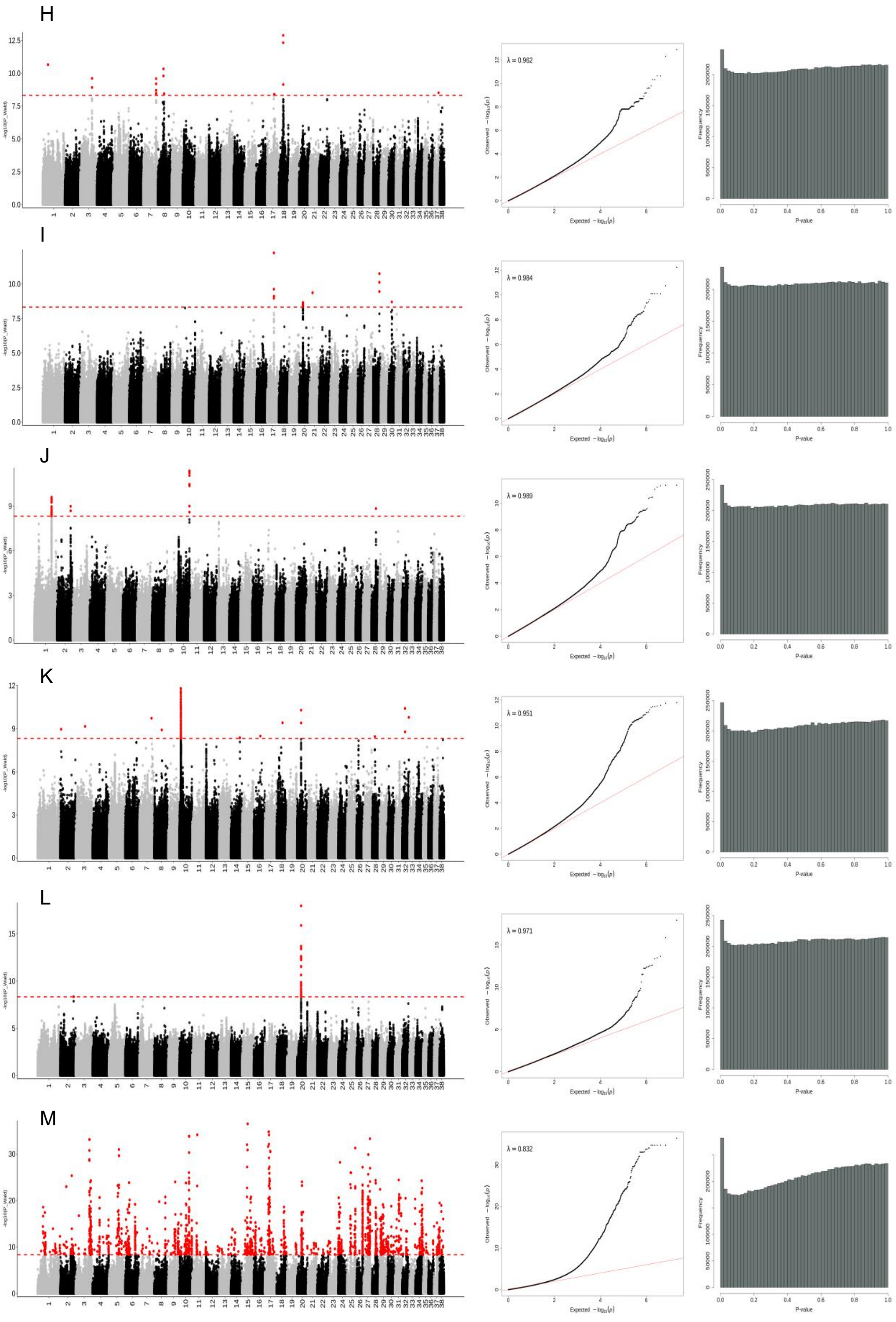

Fig. S6. GWAS results for ResNet embedding features. (**A**)–(**M**) show the Manhattan plots, QQ plots, and histograms of SNP p-value distributions for features R1–R13.

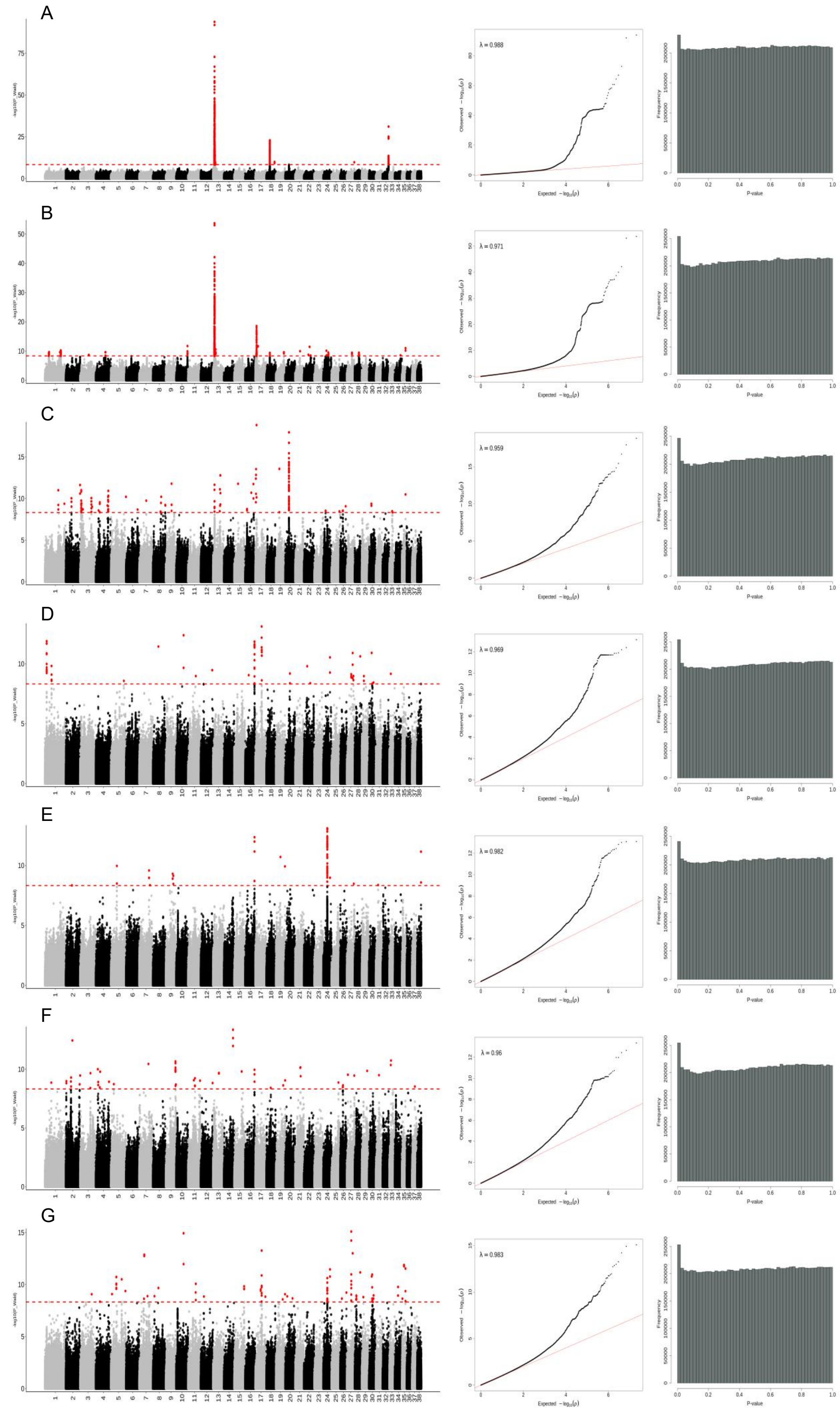
A
λ = 0.988
B
λ = 0.971
C
λ = 0.959
D
λ = 0.969
E
λ = 0.982
F
λ = 0.96
G
λ = 0.983
-log10(P_Wald)
Expected −log10(p)
Observed −log10(p)
Frequency
P-value

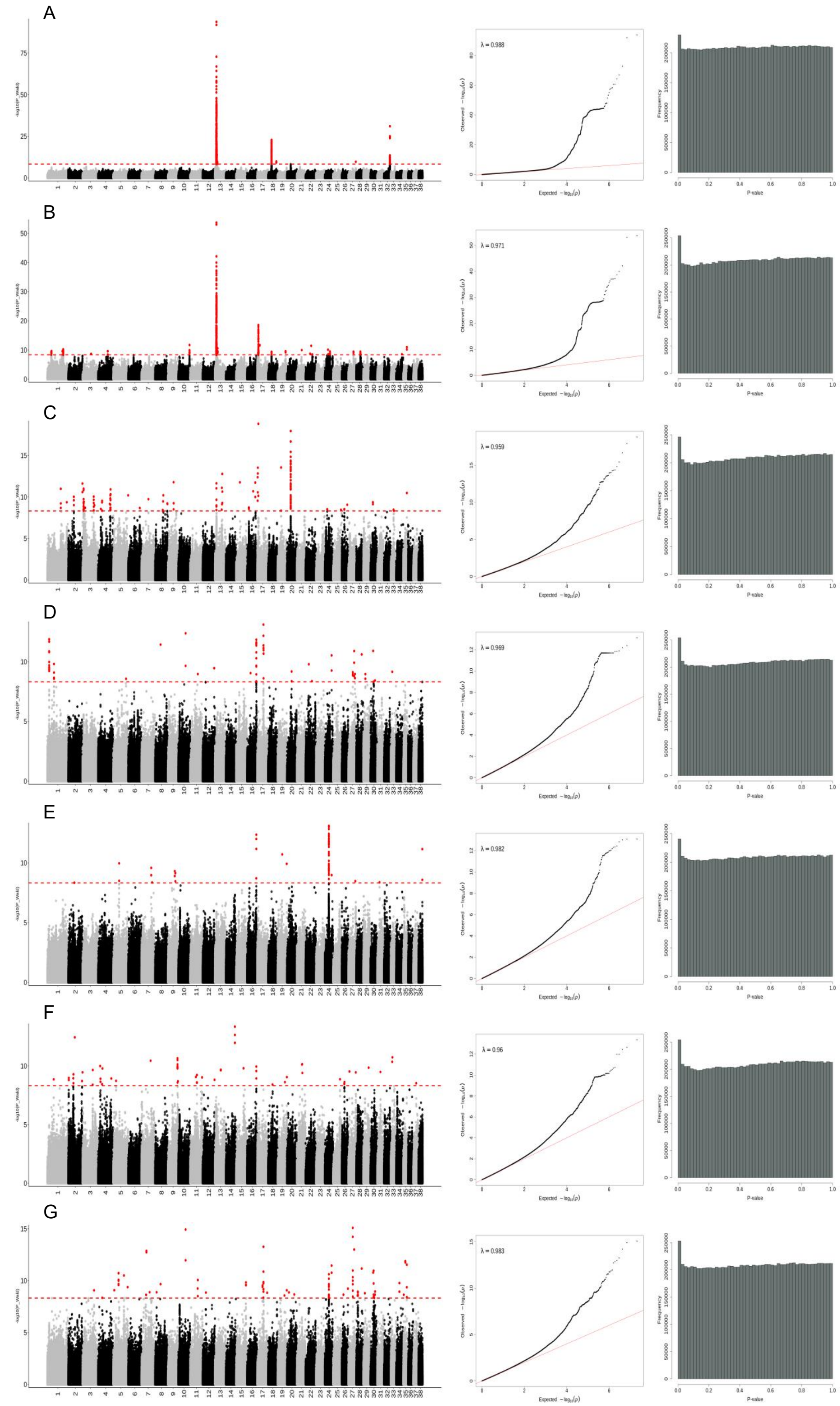

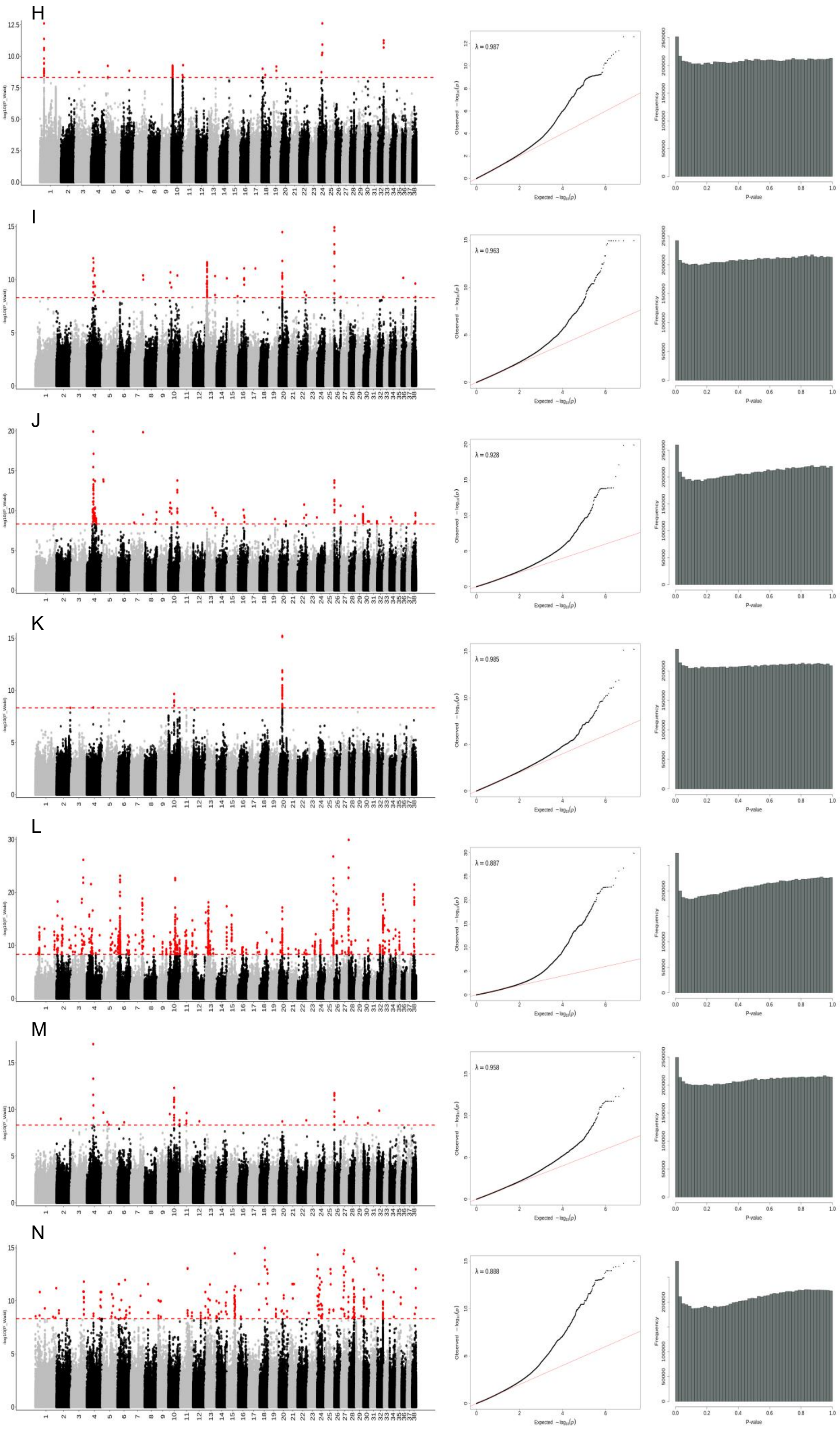
H
λ = 0.987
I
λ = 0.963
J
λ = 0.928
K
λ = 0.985
L
λ = 0.887
M
λ = 0.958
N
λ = 0.888
Expected −log10(p)
Observed −log10(p)
Frequency
P-value

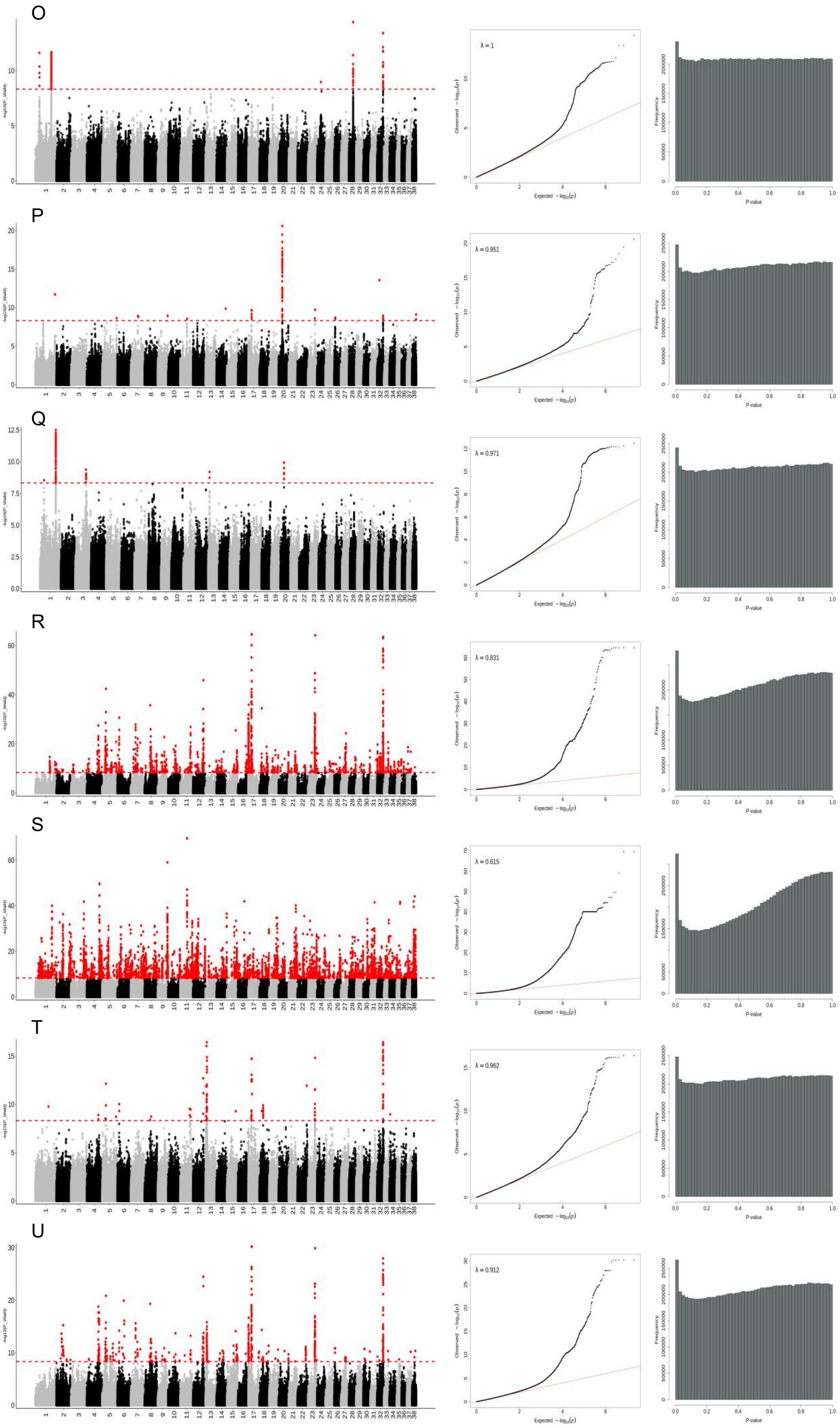

O
λ = 1
P
λ = 0.951
Q
λ = 0.971
R
λ = 0.831
S
λ = 0.615
T
λ = 0.962
U
λ = 0.912
Frequency
P-value

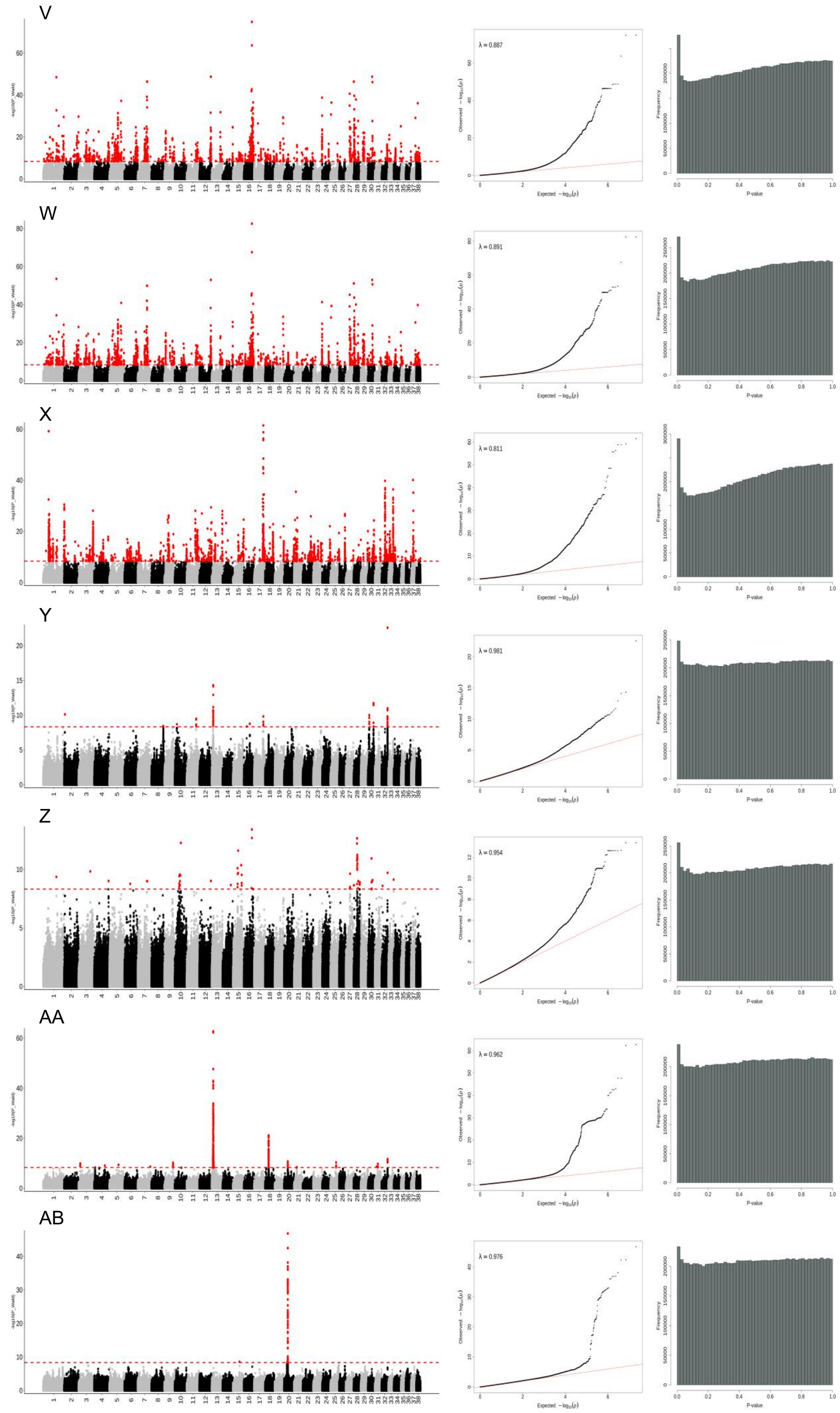

V
λ = 0.887
W
λ = 0.891
X
λ = 0.811
Y
λ = 0.981
Z
λ = 0.954
AA
λ = 0.962
AB
λ = 0.976

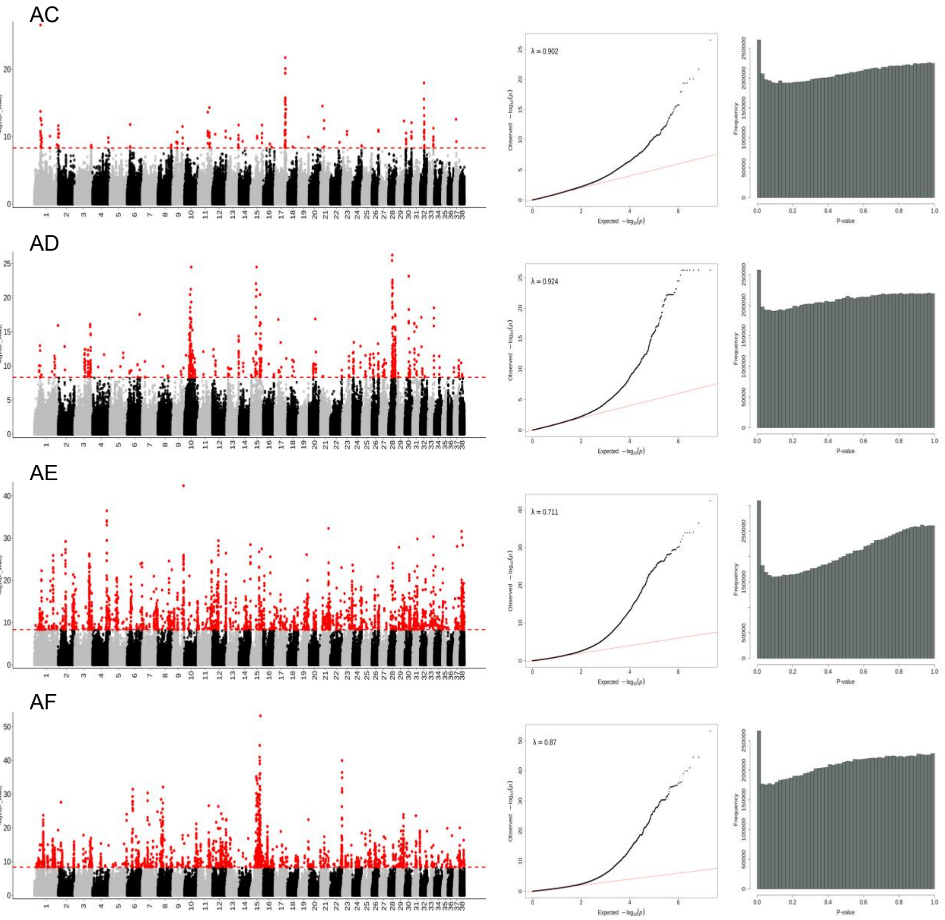

Fig. S7. GWAS results for ViT embedding features. (**A**)–(**AF**) show the Manhattan plots, QQ plots, and histograms of SNP p-value distributions for features V1-V32.

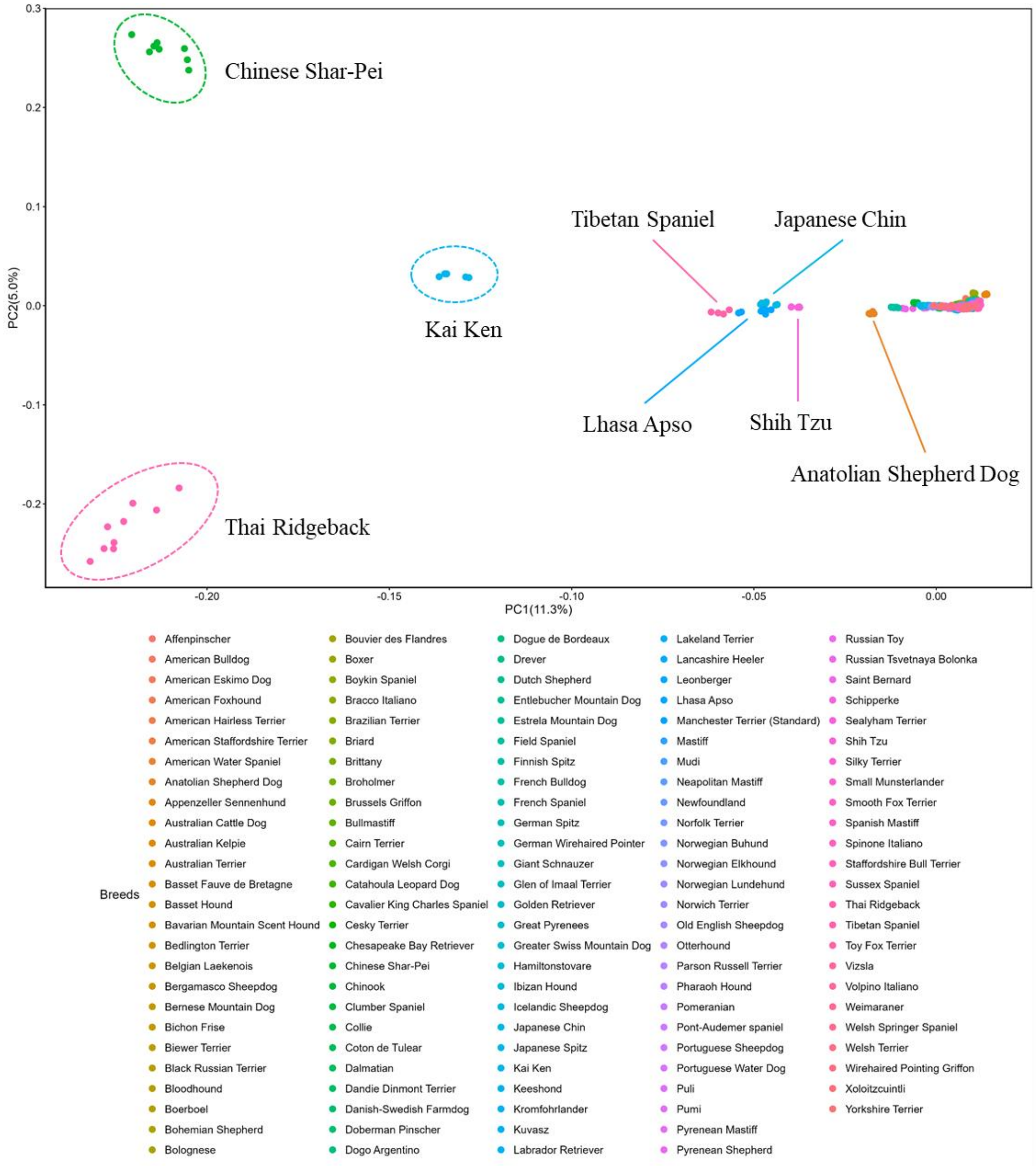

Fig. S8. Principal component analysis (PCA) of genome-wide SNP data.

Table S1. Photo credits and licensing information. Images are listed in the order in which they appear in the figures, proceeding from left to right within each row and from the top row to the bottom row.

Figure	Image	Photographer	Source	License	License Link
Fig. 1A	Danish/Swedish_farmdog	Hans863	Wikimedia Commons	CC BY-SA 3.0	https://creativecommons.org/licenses/by-sa/3.0/
Fig. 3A	Bolognese_Dog	Mrtibbs1999	Wikimedia Commons	CC BY-SA 4.0	https://creativecommons.org/licenses/by-sa/4.0
Fig. 3A	Bichon_Frise	Bernt Fransson	Wikimedia Commons	CC BY-SA 4.0	https://creativecommons.org/licenses/by-sa/4.0
Fig. 3A	Xoloitzcuintli	Maikemo	Wikimedia Commons	CC BY-SA 3.0	https://creativecommons.org/licenses/by-sa/3.0/
Fig. 3A	Peruvian_Hairless	Ferjuschin	Wikimedia Commons	CC BY-SA 4.0	https://creativecommons.org/licenses/by-sa/4.0
Fig. 3A	Coton_de_Tulear	Немања 93	Wikimedia Commons	CC BY-SA 4.0	https://creativecommons.org/licenses/by-sa/4.0
Fig. 3A	Dandie_Dinmont_Terrier	Canarian	Wikimedia Commons	CC BY-SA 4.0	https://creativecommons.org/licenses/by-sa/4.0
Fig. 3A	American_Hairless_Terrier	Sergey Akishev	Wikimedia Commons	CC BY-SA 3.0	https://creativecommons.org/licenses/by-sa/3.0/
Fig. 3A	Pharaoh_Hound	Desaix83 (after Ple2000)	Wikimedia Commons	CC BY-SA 3.0	https://creativecommons.org/licenses/by-sa/3.0/
Fig. 3A	Old_English_Sheepdog	Mona	Wikimedia Commons	CC BY-SA 3.0	https://creativecommons.org/licenses/by-sa/3.0/
Fig. 3A	Shih_Tzu	Wawri	Wikimedia Commons	CC BY-SA 2.5	https://creativecommons.org/licenses/by-sa/2.5
Fig. 3A	Boxer	Coalsi	Wikimedia Commons	CC BY-SA 3.0	https://creativecommons.org/licenses/by-sa/3.0/
Fig. 3A	Ibizan_Hound	Colin West	Wikimedia Commons	CC BY-SA 3.0	https://creativecommons.org/licenses/by-sa/3.0/

Fig. 3A	Lhasa_Apso	Lcfrederico	Wikimedia Commons	CC BY-SA 4.0	https://creativecommons.org/licenses/by-sa/4.0
Fig. 3A	Briard	Jtreier	Wikimedia Commons	CC BY-SA 3.0	https://creativecommons.org/licenses/by-sa/3.0/
Fig. 3A	Staffordshire_Bull_Terrier	mrs staffies	Wikimedia Commons	Public domain	https://creativecommons.org/publicdomain/mark/1.0/
Fig. 3A	Polish_Greyhound	Pribojchartpolski	Wikimedia Commons	CC BY-SA 4.0	https://creativecommons.org/licenses/by-sa/4.0
Fig. 3A	American_Bulldog	Householdera	Wikimedia Commons	CC BY-SA 3.0	https://creativecommons.org/licenses/by-sa/3.0/
Fig. 3A	American_Hairless_Terrier	Sergey Akishev	Wikimedia Commons	CC BY-SA 3.0	https://creativecommons.org/licenses/by-sa/3.0/
Fig. 3A	American_Akita	Edwin Campos	Wikimedia Commons	CC BY-SA 4.0	https://creativecommons.org/licenses/by-sa/4.0
Fig. 3A	Bernese_Mountain_Dog	AnetaAp	Wikimedia Commons	CC BY-SA 4.0	https://creativecommons.org/licenses/by-sa/4.0
Fig. 3A	Bergamasco_Sheepdog	Luigi Guidobono Cavalchini	Wikimedia Commons	CC BY 3.0	https://creativecommons.org/licenses/by/3.0
Fig. 3A	Coton_de_Tulear	Немања 93	Wikimedia Commons	CC BY-SA 4.0	https://creativecommons.org/licenses/by-sa/4.0
Fig. 3A	Boerboel	Jln115	Wikimedia Commons	CC BY-SA 4.0	https://creativecommons.org/licenses/by-sa/4.0
Fig. 3A	Broholmer	WLVB	Wikimedia Commons	CC BY-SA 4.0	https://creativecommons.org/licenses/by-sa/4.0
Fig. 3A	American_Eskimo_Dog	Zack Tanner	Wikimedia Commons	CC BY-SA 3.0	https://creativecommons.org/licenses/by-sa/3.0/
Fig. 3A	Cairn_Terrier	Solazar	Wikimedia Commons	CC BY-SA 3.0	https://creativecommons.org/licenses/by-sa/3.0/
Fig. 3A	Silky_Terrier	Ofsilkysdream	Wikimedia	CC BY-SA 3.0	https://creativecommons.org/

			Commons		licenses/by-sa/3.0/
Fig. 3A	Yorkshire_Terrier	Bc. Kateřina Filipi	Wikimedia Commons	CC BY-SA 3.0	https://creativecommons.org/licenses/by-sa/3.0/
Fig. 3B	French_Bulldog	Totegnac	Wikimedia Commons	CC BY-SA 4.0	https://creativecommons.org/licenses/by-sa/4.0
Fig. 3B	Neapolitan_Mastiff	Bramans	Wikimedia Commons	CC BY-SA 4.0	https://creativecommons.org/licenses/by-sa/4.0
Fig. 3B	Basset_Hound	Pohled 111	Wikimedia Commons	CC BY-SA 4.0	https://creativecommons.org/licenses/by-sa/4.0
Fig. 3B	Basset_Artesien_Normand	Bernt Sønvisen	Wikimedia Commons	CC BY 2.0	https://creativecommons.org/licenses/by/2.0
Fig. 3B	Bedlington_Terrier	David Owsiany	Wikimedia Commons	CC BY-SA 4.0	https://creativecommons.org/licenses/by-sa/4.0
Fig. 3B	Dogo_Canario	MalleMientje	Wikimedia Commons	CC BY 4.0	https://creativecommons.org/licenses/by/4.0
Fig. 3B	Finnish_Hound	EtäKärppä	Wikimedia Commons	CC BY-SA 3.0	https://creativecommons.org/licenses/by-sa/3.0/
Fig. 3B	American_Foxhound	Ltshears	Wikimedia Commons	CC BY-SA 3.0	https://creativecommons.org/licenses/by-sa/3.0/
Fig. 3B	Whippet	Sagaciousphil	Wikimedia Commons	CC BY-SA 3.0	https://creativecommons.org/licenses/by-sa/3.0/
Fig. 3B	Galgo_Espanol	Mravlja Matjaz	Wikimedia Commons	CC BY-SA 4.0	https://creativecommons.org/licenses/by-sa/4.0
Fig. 3B	Chinese_Shar-Pei	UpdOWN	Wikimedia Commons	CC BY-SA 3.0	https://creativecommons.org/licenses/by-sa/3.0/
Fig. 3B	Golden_Retriever	DustyBear1234	Wikimedia Commons	CC BY-SA 4.0	https://creativecommons.org/licenses/by-sa/4.0
Fig. 3B	Magyar_Agar	Ibolya	Wikimedia Commons	CC BY 2.0	https://creativecommons.org/licenses/by/2.0
Fig. 3B	Toy_Fox_Terrier	Terry Best	Wikimedia	CC BY-SA 3.0	https://creativecommons.org/

			Commons		licenses/by-sa/3.0/
Fig. 3B	Bavarian_Mountain_Scent_Hound	Antham1616	Wikimedia Commons	CC BY-SA 4.0	https://creativecommons.org/licenses/by-sa/4.0
Fig. 3B	Alpine_Dachsbracke	Svenska Mässan	Wikimedia Commons	CC BY 2.0	https://creativecommons.org/licenses/by/2.0

Table S2. PERMANOVA results for the association between phenotypic traits and breed-level distance matrix.

Model	Variable	N	Df	SumOfSqs	F.Model	R^2	p-value
ResNet	Coat length	127	1	523.01	34.873	0.21813	0.001
	Erect ear	128	1	106.22	5.7937	0.04718	0.002
	Weight	127	1	103.36	5.6314	0.04311	0.001
	Height	126	1	122.13	6.6986	0.05125	0.001
	Weight/Height	126	1	99.94	5.4283	0.04194	0.002
	EV	61	1	94.93	5.3128	0.08261	0.002
	CI	61	1	19.09	0.9967	0.01661	0.369
ViT	Coat length	127	1	470.00	18.106	0.12652	0.001
	Erect ear	128	1	152.8	5.3574	0.04379	0.001
	Weight	127	1	130.90	4.5646	0.03523	0.002
	Height	126	1	148.30	5.1958	0.04022	0.001
	Weight/Height	126	1	117.60	4.0858	0.03190	0.002
	EV	61	1	101.69	3.4136	0.05469	0.003
	CI	61	1	53.96	1.7633	0.02902	0.064

Table S3. Allele frequencies of 5 variants across dog breeds.

Group	chr20:22064179	chr32:35494497	chr24:23906214	chr18:20817004	chr18:48871585
Affenpinscher	0	0.1	0.7	0	1
American Bulldog	1	0	0	0	1
American Eskimo Dog	1	0.75	0.5833335	0	1
American Foxhound	1	0	0	0	1
American Hairless Terrier	0.7	0	0.1	0	1
American Staffordshire Terrier	0.6	0.1	0	0	1
American Water Spaniel	0	1	0.4166665	0	1
Anatolian Shepherd Dog	0.3333335	0.25	0	0	0.9166665
Appenzeller Sennenhund	1	0.107143	0	0	1
Australian Cattle Dog	1	0	0	0	1
Australian Kelpie	0	0	0	0	1
Australian Terrier	0	0	0	0.9	0.5
Basset Fauve de Bretagne	0	0.1	0	0.4	0.5
Basset Hound	1	0	0	0.5714285	0.5
Bavarian Mountain Scent Hound	0	0	0	0	1
Bedlington Terrier	0	0	0	0	1
Belgian Laekenois	0.1	0.1	0	0.1	1
Bergamasco Sheepdog	0.0833335	1	0.0833335	0	0.9166665
Bernese Mountain Dog	1	1	0	0	1
Bichon Frise	0.8333335	1	0	0.6666665	0.5
Biewer Terrier	1	0.9166665	0	1	0.5
Black Russian Terrier	0	1	0.2	0	1
Bloodhound	0	0	0	0	1
Boerboel	0	0	0	0	0.875
Bohemian Shepherd	0	1	0.0833335	0	0.5
Bolognese	0.8	1	0.2	0.6	0.6
Bouvier des Flandres	0	1	0	0	1
Boxer	0.3	0	0	0	1
Boykin Spaniel	0	1	0.1	0	1
Bracco Italiano	0.9166665	0	0	0	1
Brazilian Terrier	1	0	0	0	1
Briard	0	1	0	0	1
Brittany	1	1	0	0	1
Broholmer	0	0	0.0833335	0	1
Brussels Griffon	0	0	0	0	1
Bullmastiff	0	0	0	0	1
Cairn Terrier	0	0	0	1	0.5
Cardigan Welsh Corgi	1	0.1	0	1	0.5
Catahoula Leopard Dog	0	0	0	0	1
Cavalier King Charles Spaniel	0.6	1	0	0	1
Cesky Terrier	0	0	0	1	0.4
Chesapeake Bay Retriever	0.1	0	0.2	0	1
Chinese Shar-Pei	0.0625	0.0625	0	0	0.75
Chinook	0.1666665	0.0833335	0	0	0.8333335
Clumber Spaniel	1	1	0	0	1

Collie	1	0.653846	0	0	1
Coton de Tulear	1	1	0.125	1	0.5
Dalmatian	1	0	0	0	1
Dandie Dinmont Terrier	0	0	0	0.9166665	0.5
Danish-Swedish Farmdog	1	0.0833335	0	0	1
Doberman Pinscher	0	0	0	0	1
Dogo Argentino	1	0	0	0	1
Dogue de Bordeaux	0	0	0	0	1
Drever	1	0.0833335	0	0.9166665	0.5
Dutch Shepherd	0	0.3	0	0	0.9
Entlebucher Mountain Dog	1	0.0555555	0	0	1
Estrela Mountain Dog	0.1666665	1	0	0	1
Field Spaniel	0.1	1	0	0.1	1
Finnish Spitz	0.0833335	0	0	0	1
French Bulldog	0.5714285	0.0714285	0.0714285	0	1
French Spaniel	1	1	0	0	1
German Spitz	0.5833335	0.9166665	0.1666665	0	0.8333335
German Wirehaired Pointer	0.9	0	0.2	0	0.9
Giant Schnauzer	0	0.3333335	0.5833335	0	0.9166665
Glen of Imaal Terrier	0	0	0	1	0.5
Golden Retriever	0	1	0.1666665	0	1
Great Pyrenees	1	1	0	0	1
Greater Swiss Mountain Dog	1	0.05	0	0	1
Hamiltonstovare	1	0	0	0	1
Ibizan Hound	1	0	0	0	1
Icelandic Sheepdog	0.6666665	0.8333335	0.0833335	0	1
Japanese Chin	1	1	0	0	1
Japanese Spitz	1	1	0.2083335	0	0.7083335
Kai Ken	0	0	0	0	0.8
Keeshond	0	1	0	0	1
Kromfohrlander	1	0.2142855	0.2142855	0	1
Kuvasz	0.4285715	1	0.2857145	0	1
Labrador Retriever	0	0.0833335	0.1666665	0	1
Lakeland Terrier	0.0833335	0	0	0	1
Lancashire Heeler	0	0	0	0.5	0.5
Leonberger	0	1	0	0	0.9545455
Lhasa Apso	0.1875	1	0	0.6875	0.5
Manchester Terrier (Standard)	0	0	0	0.111111	1
Mastiff	0	0	0	0	1
Mudi	0	1	0.9	0	0.8
Neapolitan Mastiff	0	0	0.1	0	1
Newfoundland	0.3333335	1	0.6666665	0	1
Norfolk Terrier	0	0.0714285	0	1	0.5
Norwegian Buhund	0.5	0	0	0	1
Norwegian Elkhound	0	0	0	0	1
Norwegian Lundehund	1	0	0	0	1
Norwich Terrier	0	0.0833335	0	0.8333335	0.5

Old English Sheepdog	1	1	0	0	1
Otterhound	0.357143	0	0	0	1
Parson Russell Terrier	1	0.1	0	0	1
Pharaoh Hound	0	0	0	0	1
Pomeranian	0.0625	1	0	0	0.6875
Pont-Audemer spaniel	1	1	0	0	1
Portuguese Sheepdog	0	1	0	0	1
Portuguese Water Dog	0.4375	1	0.4375	0	1
Puli	0.0833335	1	0.75	0	0.9166665
Pumi	0.0833335	1	0.3333335	0	0.9166665
Pyrenean Mastiff	1	1	0	0	1
Pyrenean Shepherd	0	1	0	0	1
Russian Toy	0	0.6666665	0	0.1666665	0.9166665
Russian Tsvetnaya Bolonka	0.25	0.9166665	0	0.75	0.5
Saint Bernard	1	0.730769	0	0	0.923077
Schipperke	0	0	0.6666665	0	0.8333335
Sealyham Terrier	1	0	0	0.9444445	0.4444445
Shih Tzu	0.8	1	0	0.4	0.5
Silky Terrier	0	0.1666665	0	0.75	0.5
Small Munsterlander	1	1	0.8636365	0	1
Smooth Fox Terrier	1	0.0555555	0	0	1
Spanish Mastiff	0.3333335	0.1666665	0.1666665	0	1
Spinone Italiano	1	0.2857145	0	0	1
Staffordshire Bull Terrier	0.1666665	0	0	0	1
Sussex Spaniel	0	1	0.1666665	0	1
Thai Ridgeback	0	0.0555555	0.111111	0	0.9444445
Tibetan Spaniel	0.25	1	0	1	0.5
Toy Fox Terrier	1	0	0.15	0	0.85
Vizsla	0	0.0555555	0.111111	0	0.9444445
Volpino Italiano	1	1	0	0	1
Weimaraner	0	0.1	0.1	0	0.6
Welsh Springer Spaniel	1	1	0	0	1
Welsh Terrier	0	0	0	0	1
Wirehaired Pointing Griffon	1	0.1666665	0.25	0	1
Xoloitzcuintli	0.3333335	0.3333335	0.1666665	0.0833335	1
Yorkshire Terrier	0	0.5	0	0.6	0.6

Data S1. (separate file)
Sample genotype information for GWAS analysis.

Data S2. (separate file)
Breed-averaged ResNet embedding features.

Data S3. (separate file)
Breed-averaged ViT embedding features.

Data S4. (separate file)
AKC and dog cranial morphology Data.

Data S5. (separate file)
The gene list was identified by GWAS based on ResNet features.

Data S6. (separate file)
The gene list was identified by GWAS based on ViT features.
